# The mechanism of Guanxin Qiwei dropping pills target *Dubosiella* to improve atherosclerosis

**DOI:** 10.3389/fphar.2025.1633862

**Published:** 2025-07-01

**Authors:** Yuanhong Liao, Jun Li, Yuxin Li, Jing Liu, Tingting Chen, Jingkun Lu, Hongxia Li, Qian Zhang, Yuewu Wang

**Affiliations:** ^1^ School of Pharmacy, Inner Mongolia Medical University, Hohhot, Inner Mongolia Autonomous Region, China; ^2^ New Drug Screening Engineering Research Center, Inner Mongolia Medical University, Hohhot, Inner Mongolia Autonomous Region, China; ^3^ School of Basic Medical Sciences, Inner Mongolia Medical University, Hohhot, Inner Mongolia Autonomous Region, China

**Keywords:** atherosclerosis, Guanxin Qiwei dropping pills, metabolomics, gut microbiota, *Dubosiella*

## Abstract

**Objective:**

To investigate the therapeutic effects of Guanxin Qiwei dropping pills (GXQW) on atherosclerosis (AS) and to delineate the mechanisms underlying these effects.

**Methods:**

First, the chemical constituents of GXQW were identified using liquid chromatography-mass spectrometry (LC-MS). In addition, 15 batches of GXQW were used for fingerprint determination. Subsequently, an ApoE^−/−^ mouse model of AS induced by a high-fat diet was established. Lipid deposition, plaque coverage, and collagen fiber content in the aortic arch were evaluated using Oil Red O, H&E, and Masson’s trichrome staining, respectively. Enzyme-linked immunosorbent assay (ELISA) kits were employed to quantify serum oxidative stress markers, inflammatory cytokines, and lipid profiles. Additionally, fecal samples were subjected to 16S rRNA sequencing to investigate the effects of GXQW on intestinal dysbacteriosis. Differential gut microbiota were identified at the phylum-to-genus level. Furthermore, untargeted serum metabolomics was conducted to explore the potential metabolic pathways through which GXQW ameliorated AS.

**Results:**

A total of 118 chemical constituents were identified in GXQW through database comparison. Compared to the model group, GXQW treatment attenuated lipid deposition and plaque coverage in the aortic arch and mitigated collagen depletion. Fingerprint analysis showed the consistency and stability of the quality of GXQW. Additionally, GXQW reduced total cholesterol (TC) and triglyceride (TG) levels, decreased the concentrations of inflammatory cytokines interleukin-6 (IL-6) and interleukin-1beta (IL-1β), suppressed malondialdehyde (MDA) activity, and elevated superoxide dismutase (SOD) levels. In terms of gut microbiota modulation, high-dose GXQW treatment promoted the abundance of *Bacteroidota* and decreased *Firmicutes*, particularly the *Dubosiella* genus within *Firmicutes*. KEGG pathway enrichment analysis of serum metabolites revealed that pathways associated with lipid metabolism, including Glycerophospholipid metabolism, Citric acid cycle (TCA cycle), and Arachidonic acid metabolism, were notably enriched. P-cresol sulfate (PCS) and other metabolites were identified as the potential metabolic biomarkers underlying the therapeutic effects of GXQW on AS. The correlation analysis further demonstrated a significant positive correlation between *Dubosiella* and the aforementioned metabolites.

**Conclusion:**

The findings suggest that GXQW exerts evident therapeutic effects on AS by regulating gut microbiota and serum metabolic biomarkers.

## 1 Introduction

Atherosclerosis (AS) is a severe vascular disease and the most prominent type of arteriosclerosis. Its pathological changes include arterial wall thickening, lipid deposition, and plaque formation and rupture, often accompanied by various clinical manifestations and risk factors (Yuan et al., 2024). According to the 2023 “Global Cardiovascular Disease Burden” report published by the Journal of the American College of Cardiology (JACC), global mortality due to cardiovascular diseases increased from 12.4 million in 1990 to 19.8 million in 2022 (Lei et al., 2023; Kuang et al., 2024). If not properly managed, AS can lead to vascular stenosis, occlusion, organ ischemia, dysfunction, and severe complications, such as myocardial infarction, stroke, and renal failure, thereby posing a significant threat to patients’ health and survival (Zheng et al., 2024).

Metabolic diseases, such as AS, are garnering increasing attention due to the impact of gut microbiota on metabolic dysregulation (Xu et al., 2025; Sun et al., 2023). Gut microbiota is a complex community of microorganisms that interact closely with the host. Its potential roles in promoting, preventing, and treating human diseases, particularly metabolic disorders such as obesity and AS, are progressively gaining recognition. The composition and changes in the gut microbiota directly affect the host’s physiological balance (Sun et al., 2021; Chen et al., 2025). Furthermore, the metabolic potential of gut microbiota and its by-products are regarded as critical determinants influencing the host’s immune and metabolic functions (Li et al., 2025). Given the essential role of gut microbiota and its metabolites in maintaining the host’s physiological homeostasis and metabolic equilibrium, interventions targeting these factors show substantial potential for modulating body functions and alleviating metabolic disturbances.

Traditional Chinese Medicine (TCM) has been extensively accepted and applied in clinical settings in China, serving as a promising source for the development of new drugs (Chen et al., 2023; Zheng et al., 2022; Li et al., 2023). Over the years, TCM has been reported to be effective in the treatment of AS (Shao et al., 2024). For example, Fufang Danshen Dropping Pills, a traditional TCM formula, has been shown to improve blood flow, lower blood lipid levels, and enhance microcirculation, thereby alleviating lipid deposition in the arterial walls and effectively relieving various symptoms associated with AS. It also helps increase cardiac blood supply and reduces the risk of cardiovascular events (Yu Y. et al., 2024). Another TCM formulation, Shenqi, has been shown to alleviate lower limb AS by influencing blood glucose fluctuations (Huang et al., 2024). Guanxin Qiwei dropping pills (GXQW), a TCM compound composed of seven medicinal botanical drugs, including *Myristicae semen* (dried seed kernels of *Myristica fragrans* Houtt.), *Choerospondiatis fructus* (dried fruits of *Choerospondias axillaris* (Roxb.) B.L.Burtt and A.W.Hill), *Santali albi lignum* (dried heartwood of *Santalum album* L.), *Codonopisis radix* (dried roots of *Codonopsis pilosula* (Franch.) Nannf.), *Kaempferiae Rhizoma* (dried rhizomes of *Kaempferia galanga* L.), *Dalbergiae odoriferae lignum* (dried heartwood of *Dalbergia odorifera* T.C.Chen) and *Hippophae Fructus* (dried fruits of *Hippophae rhamnoides* L.). It is recorded in the Mongolian Medicine Volume of the Drug Standards of the Ministry of Health of the People’s Republic of China. GXQW is primarily used in treating coronary heart disease, irritability, palpitations, and angina (Liao et al., 2025; Yu Z. et al., 2024). *Codonopisis radix* serves as the principal ingredient, containing various active components that promote blood circulation, reduce platelet aggregation, improve microcirculation, prevent thrombosis, and exhibit antioxidant, vasodilatory, and anti-inflammatory effects (Sun et al., 2021; Chen et al., 2025). Among these components, salvianolic acid B has been demonstrated to possess anti-AS and antioxidant pharmacological activities (Li et al., 2025), while cryptotanshinone has shown properties such as anti-ischemic myocardial injury and suppression of inflammatory responses (Chen et al., 2023). Additionally, the other botanical drugs in the formula, such as *Santali albi lignum*, can relieve pain and regulate qi; *Dalbergiae odoriferae lignum* has stasis-resolving and hemostatic effects; *Myristicae semen* exerts a warming effect on the middle Jiao and facilitates Qi circulation; *Choerospondiatis fructus*, *Kaempferiae Rhizoma*, and *Hippophae Fructus* can promote blood circulation and resolve blood stasis (Zheng et al., 2022; Li et al., 2023; Shao et al., 2024; Yu Y. et al., 2024; Huang et al., 2024; Liao et al., 2025; Yu Z. et al., 2024). However, the specific mechanisms through which GXQW improves AS remain under debate.

In response to this, this study first identified the chemical components of GXQW through liquid chromatography-mass spectrometry (LC-MS). Following this, an ApoE^−/−^ mouse model of AS induced by a high-fat diet was established. Lipid accumulation, plaque coverage, and collagen content in the aortic arch were assessed using Oil Red O, H&E, and Masson staining. Additionally, ELISA kits were used to measure serum oxidative stress, inflammatory cytokines, and blood lipid levels. Furthermore, 16S rRNA sequencing of feces was conducted to investigate the effect of GXQW on gut microbiota dysbiosis, with differential microbiota being selected at the phylum-to-genus level. Untargeted serum metabolomics was also employed to elucidate the potential mechanisms of GXQW in improving AS, with differential serum metabolites identified based on *P* < 0.05, VIP > 1, and Log_2_FoldChange > 2. Finally, a correlation analysis between characteristic gut microbiota and differential metabolites was conducted. This study aims to clarify the mechanisms through which GXQW improves AS, establishing a theoretical basis for future strategies aimed at regulating the gut microbiota through TCM to improve AS.

## 2 Materials and methods

### 2.1 Experimental materials

Individual botanical drugs of GXQW were sourced from the markets in Sichuan and Anhui provinces (China). Enzyme-linked immunosorbent assay (ELISA) kits were purchased from Abbkine Biotechnology Co., Ltd. (China). For detailed information, please refer to Supplementary Table S1.

### 2.2 Animals

ApoE^−/−^ mice were purchased from Sipeifu Biotechnology Co., Ltd. (Beijing, China) (License No. SCXK (Beijing) 2019-0010) and housed at the Animal Experiment Center of Inner Mongolia Medical University (SPF, License No. SYXK (Meng) 2020-0003). At 6 weeks of age, Male mice (n = 12 per group).

### 2.3 Preparation of GXQW

Guanxin Qiwei dropping pills (GXQW), a TCM compound composed of seven medicinal botanical drugs, including *Myristicae semen* (batch number: 220101-02, dried seed kernels of *M. fragrans* Houtt.), *Choerospondiatis fructus* (batch number: 170901, dried fruits of *C. axillaris* (Roxb.) B.L.Burtt and A.W.Hill), *Santali albi lignum* (batch number: 221001, dried heartwood of *S. album* L.), *Codonopisis radix* (batch number: 230601, dried roots of *C. pilosula* (Franch.) Nannf.), *Kaempferiae Rhizoma* (batch number: A220208, dried rhizomes of *K. galanga* L.), *Dalbergiae odoriferae lignum* (batch number: 210401, dried heartwood of *D. odorifera* T.C.Chen) and *Hippophae Fructus* (batch number: 710220901, dried fruits of *H. rhamnoides* L.). It is recorded in the Mongolian Medicine Volume of the Drug Standards of the Ministry of Health of the People’s Republic of China. The whole plant materials of the seven botanical drug materials were obtained from their original source and the botanic identification was confirmed by Professor ShengSang Na (Inner Mongolia Medical University, Hohhot, China). The specimens were deposited at the herbarium of medicinal plants (The Center for New Drug Safety Evaluation and Research, Inner Mongolia Medical University, Roudoukou20240016, Guangzao20240017, Tanxiang20240018, Danshen20240019, Shannai20240020, Jiangxiang20240021, Shaji20240022. The detailed botanical drugs parts of GXQW are shown in Supplementary Table S2. The plant names were verified on 17 Jun 2025, from https://mpns.science.kew.org/mpns-portal (Heinrich et al., 2022).

**FIGURE 1:**
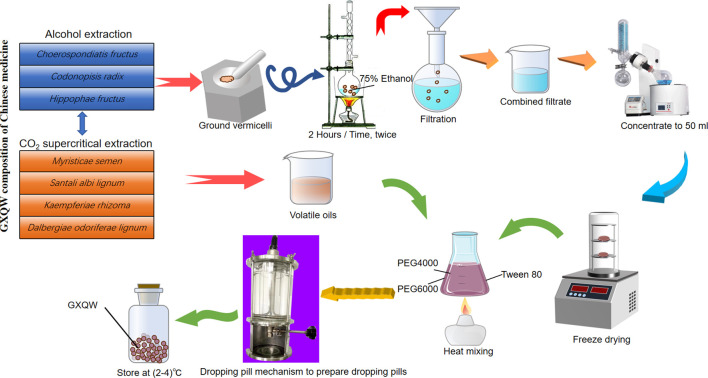
The preparation process of Guanxin Qiwei Dropping Pills GXQW.

The clinical original dosage is 6.9 g per person per day, with extraction carried out in two parts. *Choerospondiatis fructus*, *Codonopisis radix*, and *Hippophae Fructus* were combined (4.6 g) for alcohol extraction, yielding 0.98 g of extract at a 21.3% extraction rate. Meanwhile, *Myristicae semen*, *Santali albi lignum*, *Kaempferiae Rhizoma,* and *Dalbergiae odoriferae lignum* (2.3 g) underwent supercritical CO_2_ extraction, producing 0.23 g of volatile oil with a 10% extraction rate. This extract was then combined with 1.5 times polyethylene glycol and Tween 80 to prepare the drops, totaling 3.03 g. The preparation process of GXQW is shown in Figure 1. Considering the average weight of a normal adult (60 kg) and the human-to-mouse conversion factor (9.1), the equivalent dosage for mice was calculated as follows: GXQW-low dose = 3.03 g × 9.1/60 kg = 460 mg/kg, GXQW-medium dose = 3.03 g × 9.1/60 kg × 2 = 920 mg/kg and GXQW-high dose = 3.03 g × 9.1/60 kg × 4 = 1840 mg/kg. Of which 3.03 g includes 1.21 g of drug dose (0.98 g + 0.23 g), and the other 1.82 g is the weight of excipients. Therefore, the actual drug dose given to the animal should be: GXQW-low dose = 1.21 g × 9.1/60 kg = 183.52 mg/kg, GXQW-medium dose = 1.21 g × 9.1/60 kg × 2 = 367.04 mg/kg, GXQW-high dose = 1.21 g × 9.1/60 kg × 4 = 734.08 mg/kg. Therefore, the actual high dose of GXQW does not exceed 1 g/kg.

### 2.4 Detection of GXQW components

An ACQUITY UPLC I-Class HF ultra-high-performance liquid chromatography system combined with a QE high-resolution mass spectrometer was employed in the experiment. Chromatographic conditions: The chromatographic column was ACQUITY UPLC HSS T3, with a column temperature of 45°C. Mobile phase A consisted of water with 0.1% formic acid, and mobile phase B was acetonitrile, with a flow rate of 0.35 mL/min. Additionally, the PDA scanning range was between 210 and 400 nm. The elution gradients are detailed in Supplementary Table S3. Mass spectrometry conditions: The ion source was HESI. The signal collection was carried out in both positive and negative ion scanning modes. The data acquisition mode was DDA. The scan mode was Full MS/dd-MS2 (TOP 8). Specific parameters are listed in Supplementary Table S4.

### 2.5 GXQW fingerprint

#### 2.5.1 Chromatographic conditions

The chromatographic analysis was performed using a Shim-pack GIST-HP C18 column (2.1 mm × 100 mm, 3 μm). The mobile phase consisted of methanol (A) and 0.2% phosphoric acid aqueous solution (B), delivered at a flow rate of 0.8 mL/min. The column temperature was maintained at 35°C, with an injection volume of 5 μL. Detection was carried out at 254 nm. The gradient elution program was as follows: 0–8 min: 8% → 25% A; 8–20 min: 25% → 40% A; 20–30 min: 40% → 42% A; 30–40 min: 42% → 55% A; 40–45 min: 55% → 58% A; 45–60 min: 58% → 66% A; 60–75 min: 66% → 75% A; 75–78 min: 75% → 75% A; 75–78 min: 75% → 75% A; 78–82 min: 75% → 8% A; 82–90 min: 8% → 8% A.

#### 2.5.2 Establishment of GXQW fingerprint

Fifteen batches of GXQW samples were analyzed under the specified chromatographic conditions. The resulting chromatograms were converted into CDF format and imported into the 2012 version of the Chinese Medicine Chromatographic Fingerprint Similarity Evaluation System. Sample S1 of GXQW was selected as the reference chromatogram, and fingerprint analysis was performed using the median method with a time window of 0.1 min. After applying multi-point correction and marker peak alignment, the overlaid chromatograms and the reference fingerprint profile were successfully generated.

### 2.6 Animal administration and model construction

ApoE^−/−^ male mice were used as the model animals. The mice were randomly divided into a control group, a model group, a high-dose GXQW group (1840 mg/kg), a medium-dose GXQW group (920 mg/kg), a low-dose GXQW group (460 mg/kg), and a positive drug simvastatin group (2.6285 mg/kg), with 12 mice per group. Except for the control group, all groups were fed a high-fat diet for 8 weeks to induce AS. Afterward, all groups except the control and model groups were given different concentrations of GXQW and simvastatin by gavage for eight consecutive weeks. The diet and body weight of the mice were monitored throughout the experiment. All animal experiments in this study were approved by the Animal Ethics Committee of Inner Mongolia Medical University (YKD202404035).

### 2.7 Serum biochemical analysis

According to the instructions provided by the reagent kits, the levels of the following serum biochemical markers were measured: total cholesterol (TC), triglycerides (TG), low-density lipoprotein cholesterol (LDL-C), high-density lipoprotein cholesterol (HDL-C), interleukin-6 (IL-6), interleukin-1beta (IL-1β), superoxide dismutase (SOD), and malondialdehyde (MDA).

### 2.8 Histopathological analysis

At the end of the experimental period, the mice were euthanized, and the aortas were collected and fixed in 4% paraformaldehyde for histological analysis. The abdominal aorta of ApoE^−/−^ mice was dissected and subjected to Oil Red O staining. The aortic sinuses were embedded in the OCT compound, frozen, and sectioned. Afterward, Oil Red O staining, Hematoxylin-Eosin (H&E) staining, and Masson’s Trichrome staining were performed to observe lipid content, plaque coverage, and collagen fiber content. Images were captured under a stereomicroscope. The lipid area ratio was calculated using Image-Pro Plus 6.0 software.

### 2.9 Sequencing of microbiota from colonic excrement samples and data analysis

#### 2.9.1 DNA extraction and PCR amplification

Genomic DNA was extracted from the colonic content using the MagPure Soil DNA LQ Kit (Magan) according to the manufacturer’s instructions. DNA concentration and purity were determined using a NanoDrop 2000 (Thermo Fisher Scientific, United States) and agarose gel electrophoresis. The extracted DNA was stored at −20°C. PCR amplification of the 16S rRNA gene was performed using barcoded specific primers and Takara Ex Taq high-fidelity polymerase. The universal primers 343F (5′-TACGGRAGGCAGCAG-3′) and 798R (5′-AGG​GTA​TCT​AAT​CCT-3′) were used to amplify the V3-V4 hypervariable region of the 16S rRNA gene for bacterial diversity analysis.

#### 2.9.2 Library construction and sequencing

The PCR products were verified using agarose gel electrophoresis and purified using AMPure XP beads. Subsequently, they were used as templates for a second round of PCR amplification and purification. The purified second-round products were quantified using the Qubit fluorometer, and the concentration was adjusted accordingly for sequencing. Sequencing was performed using the Illumina NovaSeq 6000 platform, generating 250 bp paired-end reads.

#### 2.9.3 Bioinformatics analysis

Raw sequence data were processed using Cutadapt software to trim primer sequences. Based on DADA2 (Fan et al., 2023), The resulting qualified paired-end data were subjected to quality control analysis using QIIME2 (Yang et al., 2024) with default parameters. This process included quality filtering, denoising, merging, and chimera removal, ultimately generating representative sequences and an ASV (Amplicon Sequence Variant) abundance table. Representative sequences for each ASV were selected using the QIIME2 pipeline and subsequently taxonomically annotated by aligning against the SILVA database (version 138). Taxonomic assignment was performed using the ‘q2-feature-classifier’ plugin with default parameters. Alpha and beta diversity analyses were conducted within the QIIME2 framework. Alpha diversity, including Good’s coverage (Islam et al., 2025; Gao et al., 2024), was adopted to evaluate sample alpha diversity. Unweighted UniFrac distance matrices were computed in R and used to perform Principal Coordinates Analysis (PCoA) to assess beta diversity. Differential abundance analysis was carried out using statistical methods such as ANOVA, Kruskal-Wallis, t-tests, and Wilcoxon tests in R packages.

### 2.10 Metabolomics analysis

#### 2.10.1 Analytical conditions

The analysis was conducted using a Waters ACQUITY UPLC I-Class plus coupled with a Thermo QE high-resolution mass spectrometer. Chromatographic conditions: column is ACQUITY UPLC HSS T3 (100 mm × 2.1 mm, 1.8 µm); column temperature is 45°C; mobile phase A is water (containing 0.1% formic acid); mobile phase B is acetonitrile; flow rate is 0.35 mL/min; injection volume is 3 μL. The elution gradient is presented in Supplementary Table S5, and the mass spectrometry parameters are listed in Supplementary Table S6.

#### 2.10.2 Sample preparation

Serum samples from ApoE^−/−^ mice were added a protein precipitation reagent (methanol-acetonitrile mixture, V1:V2 = 2:1, containing internal standard at 4 μg/mL), vortexed, allowed to stand, and then centrifuged. The supernatant was collected for LC-MS analysis. Quality control (QC) samples were prepared by mixing equal volumes of all extracted samples. All reagents for extraction were pre-cooled at −20°C before use.

#### 2.10.3 Bioinformatics analysis

First, unsupervised principal component analysis (PCA) was performed to observe the overall distribution of samples and assess the stability of the analysis process. Subsequently, supervised partial least squares discriminant analysis (PLS-DA) was applied to differentiate the metabolic profiles between the groups and identify the differential metabolites.

### 2.11 Statistical analysis

Prior to analysis, all data were assessed for normality and homogeneity of variance. SPSS software was used for statistical analysis, and GraphPad Prism version 10 was used to generate graphs. For analysis of differences between the groups, we used one-way ANOVA and LSD *post hoc* tests. There was statistical significance at the *P* < 0.05 level.

## 3 Results

### 3.1 Comprehensive chemical profiling of GXQW using UPLC-QE Orbitrap MS

**FIGURE 2:**
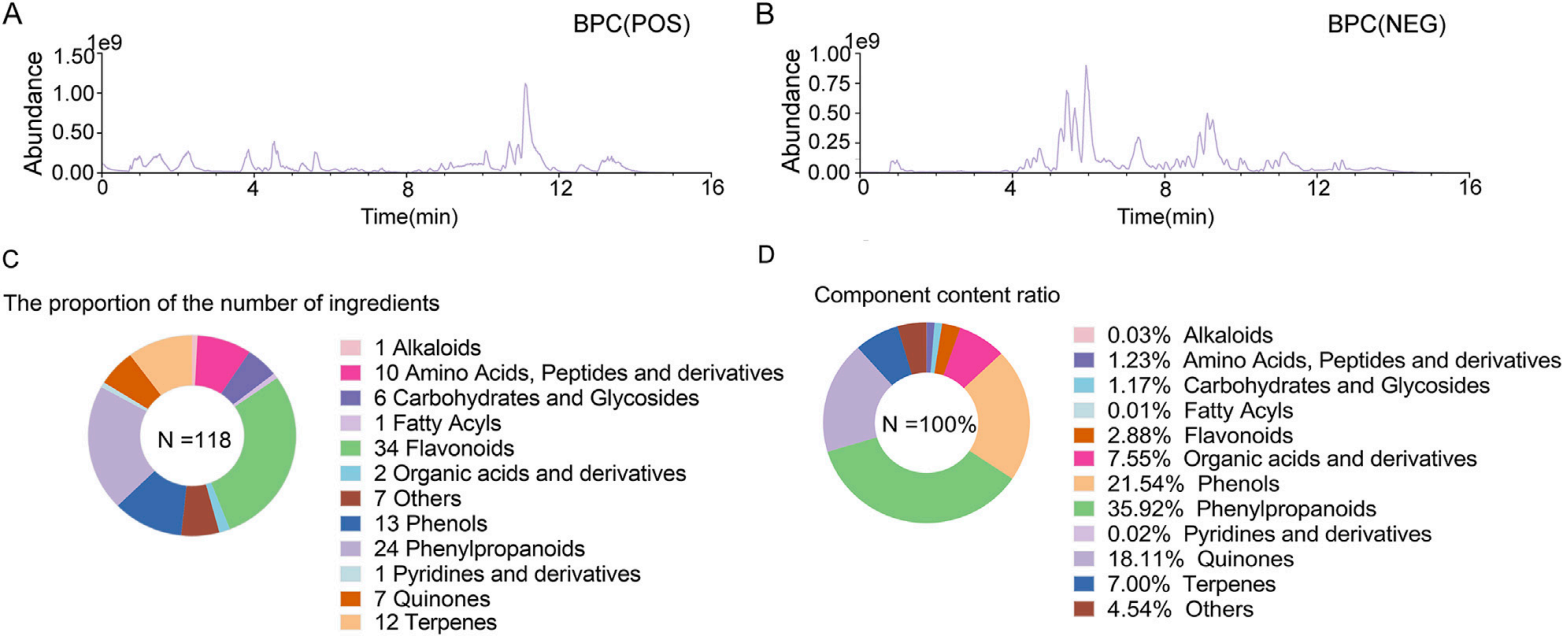
Chemical composition analysis of Guanxin Qiwei Dropping Pills (GXQW). **(A)** Base peak ion (BPC) chromatogram under positive ion mode. **(B)** BPC chromatogram under negative ion mode. **(C)** Statistics of the number of chemical components in GXQW. **(D)** Statistics of the content of chemical components in GXQW.

In order to identify chemical components, GXQW was analyzed using the ACQUITY UPLC I-Class HF coupled with a Thermo Orbitrap QE high-resolution mass spectrometer. The base peak ion (BPC) chromatograms in both positive and negative ion modes were obtained (Figures 2A,B). A total of 118 compounds from GXQW were identified by comparison with databases and references (Supplementary Table S7). Among these, flavonoids were the most abundant class, while phenylpropanoids had the highest content (Figures 2C,D).

### 3.2 The GXQW fingerprint analysis indicated a high degree of quality consistency across all 15 sample batches

**FIGURE 3:**
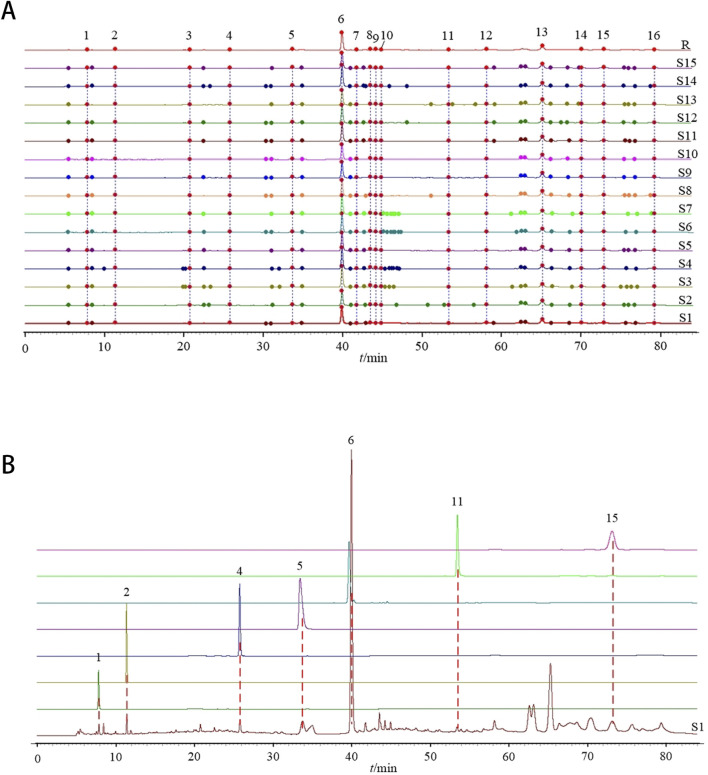
Establishment of GXQW fingerprint. **(A)** Fingerprint of 15 batches of GXQW samples. **(B)** Comparison chromatogram of single reference solution and sample (S1).

A total of 16 peaks were observed in the fingerprint chromatogram, as illustrated in Figure 3A. By comparing the chromatograms with those of individual reference standards, seven peaks were successfully identified: peak 1 as gallic acid, peak 2 as protocatechuic acid, peak 4 as kaempferol-3-O-sophoroside, peak 5 as ellagic acid, peak 6 as salvianolic acid B, peak 11 as isorhamnetin, and peak 15 as tanshinone I, as shown in Figure 3B. The similarity evaluation between the sample fingerprints and the generated reference spectrum is presented in Table 1. The similarity scores for all 15 sample batches ranged from 0.985 to 1.000, demonstrating excellent quality consistency.

**TABLE 1:** Similarity evaluation results of 15 batches of GXQW samples.

Sample no.	Similarity	Sample no.	Similarity	Sample no.	Similarity
S1	0.999	S6	0.998	S11	1.000
S2	0.985	S7	0.997	S12	0.999
S3	0.991	S8	0.993	S13	0.985
S4	0.994	S9	0.996	S14	0.990
S5	0.997	S10	0.996	S15	0.997

### 3.3 GXQW attenuates atherosclerosis and enhances plaque stability in high-fat diet-induced ApoE^−/−^ mice

The high-fat diet-induced ApoE^−/−^ mouse model was used to study AS, simulating human atherosclerotic lesions. ApoE^−/−^ mice were used to validate the therapeutic effects of GXQW.

**FIGURE 4:**
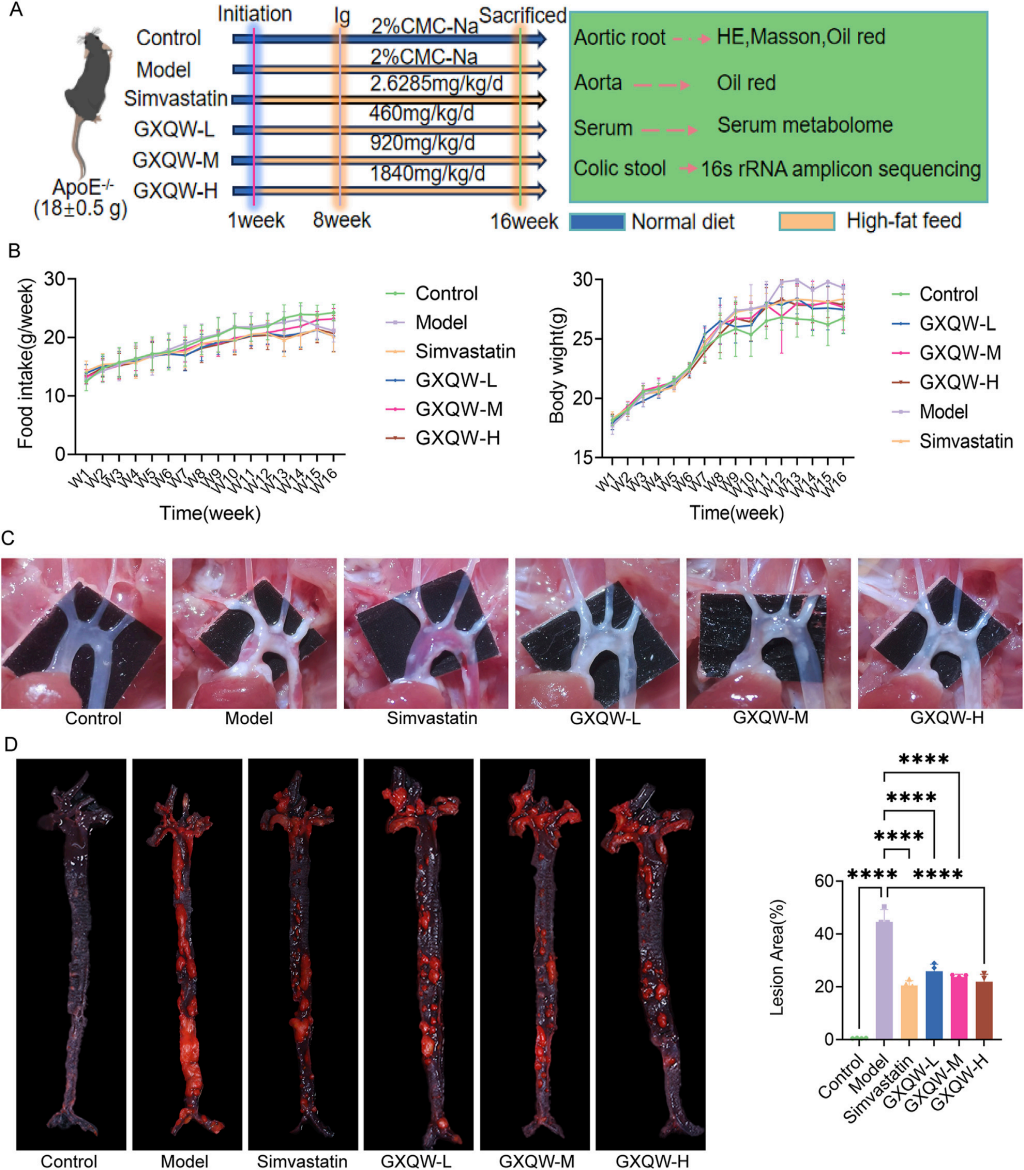
Effects of GXQW on pathological indicators of ApoE^−/−^ mice with Atherosclerosis (AS) induced by high-fat diet. **(A)** Dietary conditions of ApoE^−/−^ mice. **(B)** Body weight changes in ApoE^−/−^ mice. **(C)** Image of the aortic arch in ApoE^−/−^ mice. **(D)** Oil Red O staining for lipid detection in ApoE^−/−^ mice. Data are expressed as mean ± standard deviation (n = 7). *P < 0.05, **P < 0.01, ***P < 0.001, and ****P < 0.0001. ns: no significance.

As shown in Figure 4A, the diet and body weight of the ApoE^−/−^ mice were monitored weekly. The results indicated no significant differences in diet between the groups, suggesting that GXQW significantly inhibited body weight gain in the AS mice (Figure 4B).

**FIGURE 5:**
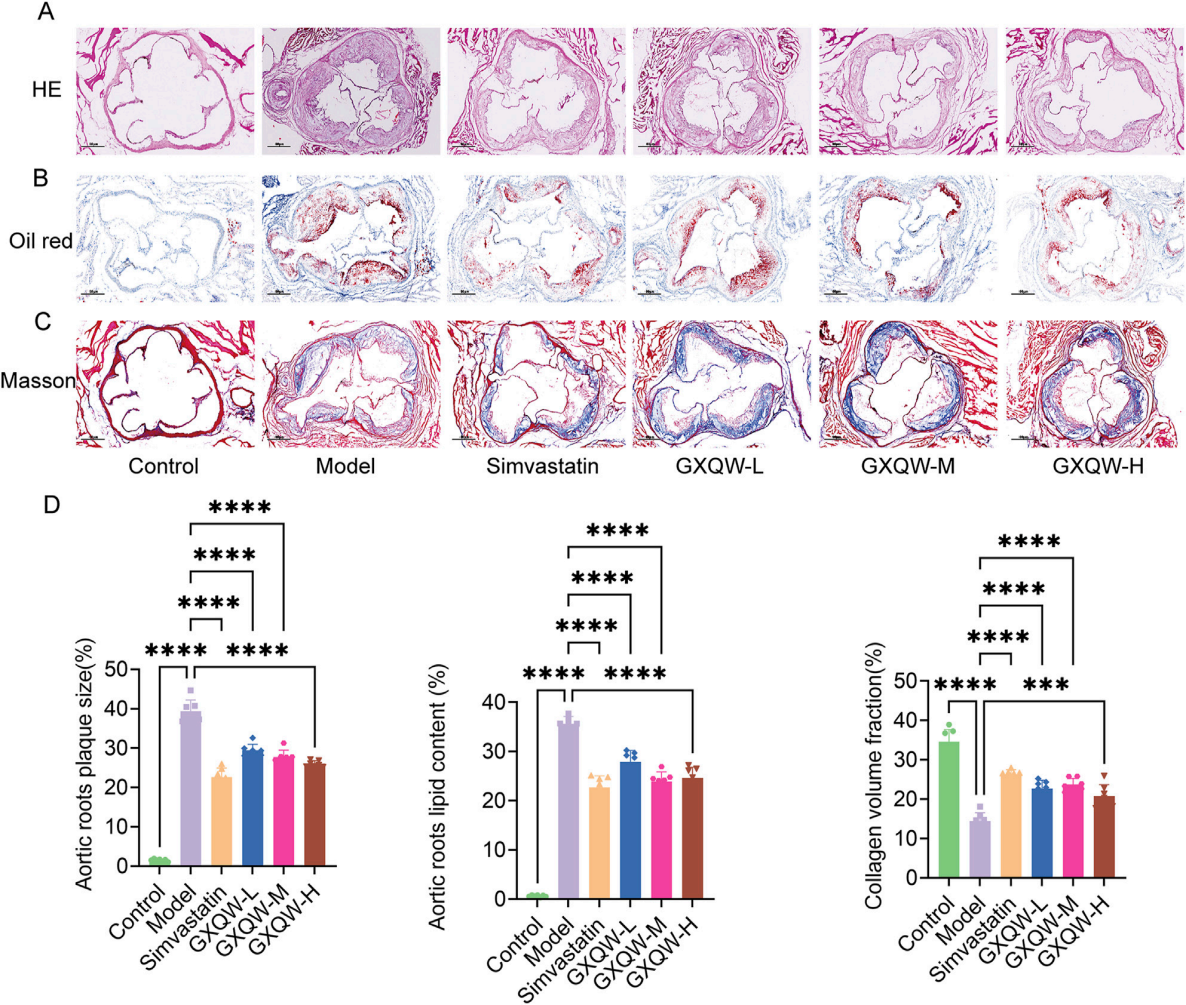
Therapeutic effects of GXQW in mice with vulnerable Atherosclerotic plaques. **(A)** H&E staining of the aortic root. **(B)** Oil Red O staining of the aortic root. **(C)** Masson’s trichrome staining of the aortic root (scale bar = 200 µm). **(D)** Quantitative analysis of lipid deposition and collagen fiber areas in cross-sections of the aortic root (mean ± standard deviation, n = 7). *P < 0.05, **P < 0.01, ***P < 0.001, and ****P < 0.0001. ns: no significance.

Furthermore, the distribution of aortic arch plaques in ApoE^−/−^ mice was directly examined, and the deposition of atherosclerotic plaques was assessed via Oil Red O staining of aortic tissues. The results are shown in Figures 4C,D. Compared to the control group, the model group exhibited extensive plaque formation, while GXQW treatment reduced plaque formation. Similarly, abundant red-stained lipid droplets were observed in the model group, which were apparently mitigated in the GXQW treatment group. These results indicated that GXQW effectively limited the accumulation of atherosclerotic plaques in ApoE^−/−^ mice induced by a high-fat diet. HE staining and Oil Red O staining of the aortic arch confirmed these findings (Figures 5A,B). Additionally, Masson’s Trichrome staining of the aortic arch revealed that GXQW increased collagen fiber content in the aorta, thus promoting plaque stability (Figures 5C,D).

### 3.4 GXQW improves lipid metabolism and reduces inflammatory cytokines in atherosclerotic mice

**FIGURE 6:**
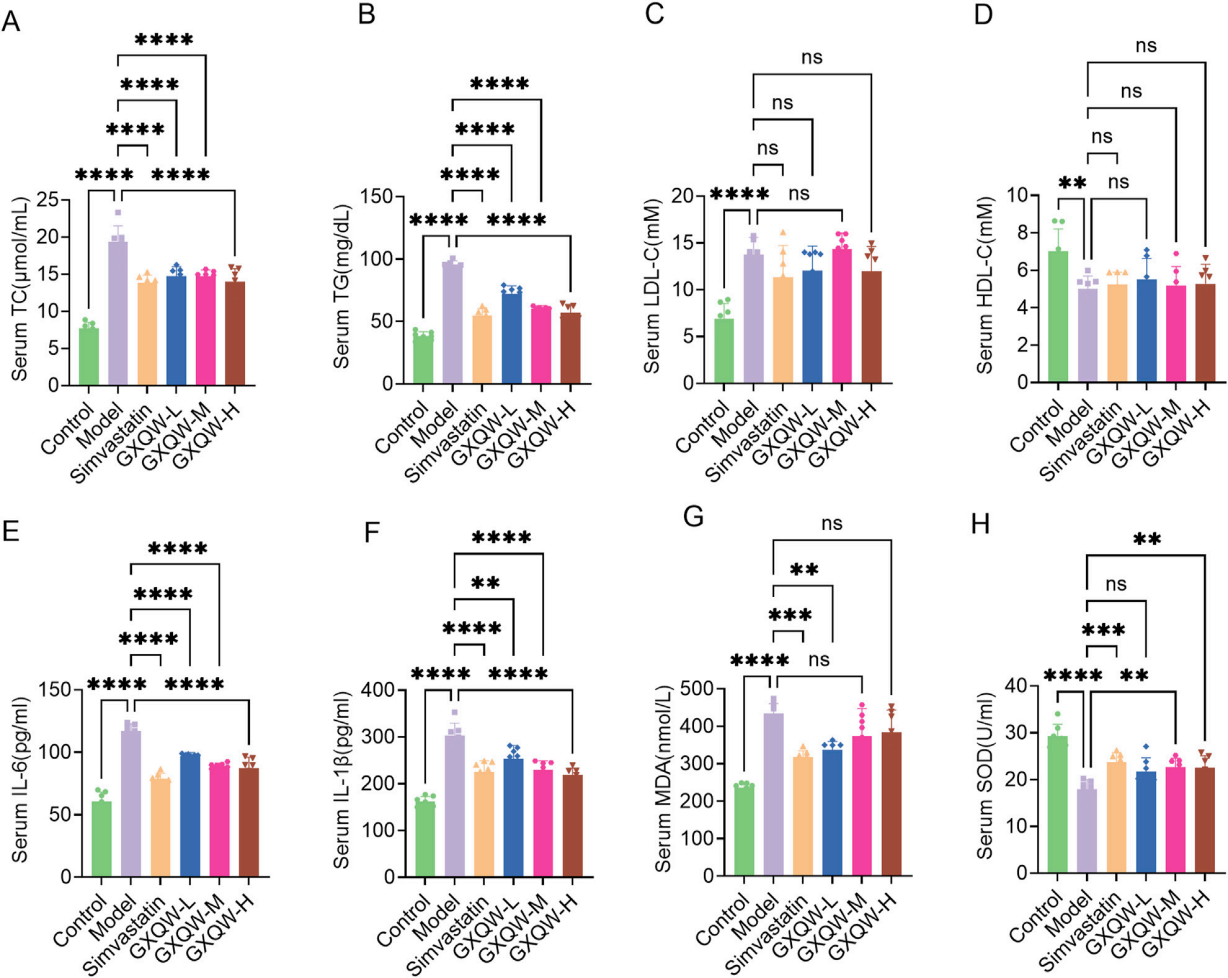
Plasma parameter analysis of ApoE^−/−^ mice treated with GXQW. **(A)** Total cholesterol (TC) levels. **(B)** Triglyceride (TG) levels. **(C)** Low-density lipoprotein cholesterol (LDL-C) levels. **(D)** High-density lipoprotein cholesterol (HDL-C) levels. **(E)** Interleukin-6 (IL-6) levels. **(F)** Interleukin-1β (IL-1β) levels. **(G)** Malondialdehyde (MDA) levels. **(H)** Superoxide dismutase (SOD) levels. Data are presented as mean ± standard deviation, n = 7. *P < 0.05, **P < 0.01, ***P < 0.001, and ****P < 0.0001. ns: no significance.

Given the association of atherosclerotic plaque growth with hypercholesterolemia, lipid levels in the blood were examined. Compared to the control group, high-fat feeding in ApoE^−/−^ mice increased the levels of TG, TC, and LDL-C and lowered HDL-C levels. Treatment with GXQW significantly reduced TC and TG levels (Figures 6A,B) but barely affected LDL-C and HDL-C levels (Figures 6C,D).

Inflammatory cytokines promote the development of AS. Therefore, the expression levels of IL-6 and IL-1β in the plasma were detected. Compared to the control group, high-fat diet-fed ApoE^−/−^ mice had elevated levels of plasma IL-6 and IL-1β, whereas GXQW treatment reduced the levels of these cytokines (Figures 6E,F).

Moreover, GXQW attenuated the accumulation of MDA, a lipid peroxidation product, and alleviated the abnormal reduction of SOD in the serum (Figures 6G,H).

### 3.5 GXQW modulates gut microbiota composition to restore microbial homeostasis in atherosclerotic mice

Considering that GXQW exhibited significant anti-atherosclerotic effects, its potential modulation of the gut microbiota in AS mice may serve as a therapeutic mechanism. Therefore, the GXQW-H group (chosen based on pharmacological analysis results) was selected for this study. The composition of the gut microbiota in the control, model, and GXQW-H groups of mice was analyzed using 16S rRNA sequencing.

**FIGURE 7:**
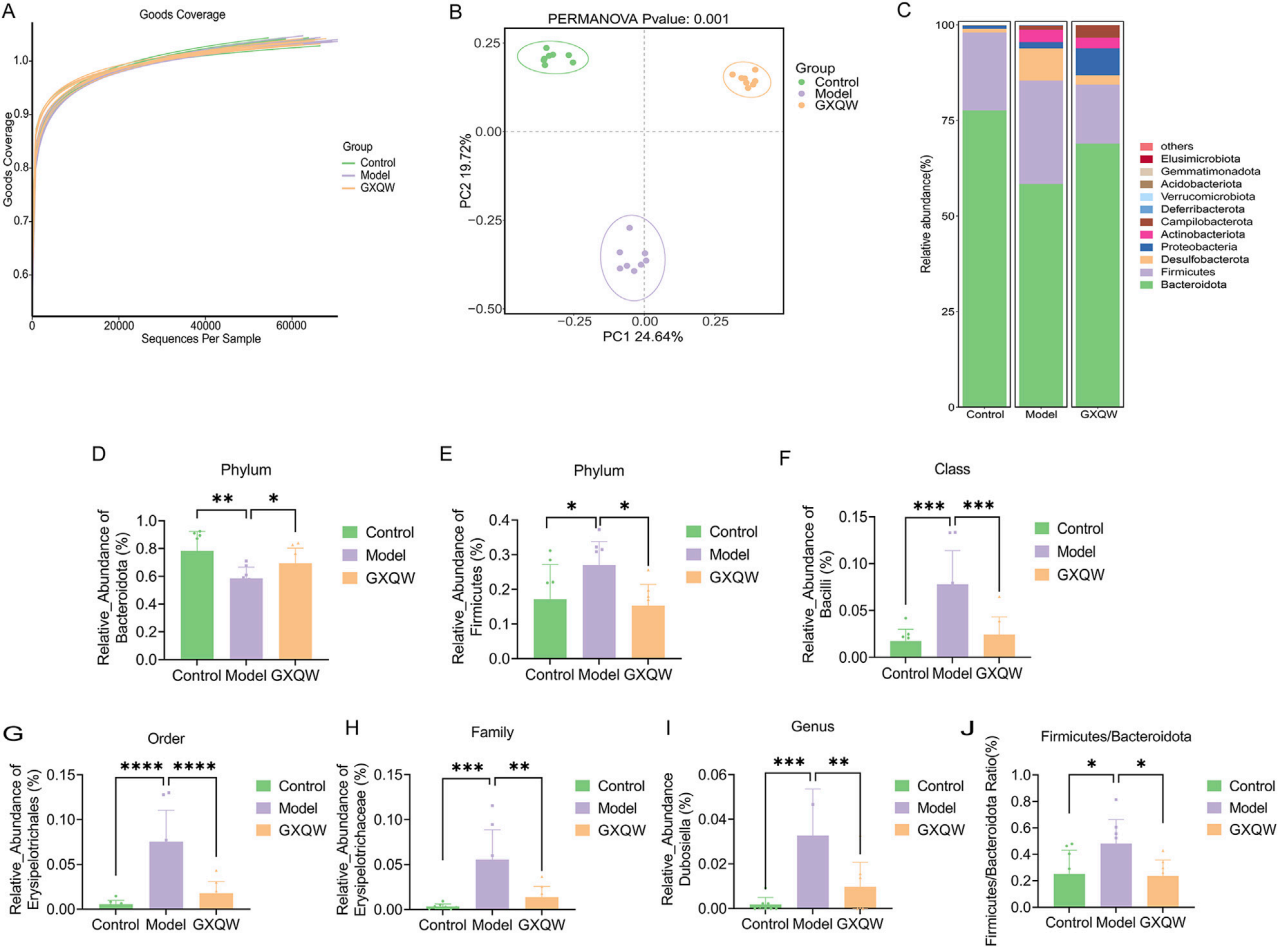
Effects of GXQW on the intestinal flora of high-fat diet-induced AS Mice. **(A)** The Good’s coverage curve of microbial diversity. **(B)** PCA score plot. **(C)** Heatmap analysis of intestinal flora in control, model, and GXQW groups. **(D)** Relative abundance of *Bacteroidota* at the phylum level in control, model, and GXQW groups. **(E)** Relative abundance of *Firmicutes* at the phylum level in control, model, and GXQW groups. **(F)** Relative abundance of *Bacilli* at the class level in control, model, and GXQW groups. **(G)** Relative abundance of *Erysiplotrichales* at the order level in control, model, and GXQW groups. **(H)** Relative abundance of *Erysipelotrichaceae* at the family level in control, model, and GXQW groups. **(I)** Relative abundance of *Dubosiella* at the genus level in control, model, and GXQW groups. **(J)** The relative abundance ratio of *Firmicutes*/*Bacteroidota* (F/B) at the phylum level in control, model, and GXQW groups. Data are presented as mean ± standard deviation, n = 8. *P < 0.05, **P < 0.01, ***P < 0.001, and ****P < 0.0001. ns: no significance.

The richness and diversity of the bacterial communities were assessed based on alpha diversity (Figure 7A). All samples exhibited a Good’s coverage index greater than 0.999, implying that the sampling depth was sufficient to capture most of the bacteria. Furthermore, significant differences in beta diversity (microbial composition and structure) were found between the groups (*P* < 0.001) (Figure 7B). These findings demonstrated that, compared to healthy mice, the gut microbiota composition in high-fat diet-induced AS mice was altered. Additionally, GXQW treatment evidently influenced the gut microbiota composition in AS mice, suggesting its potential therapeutic role in restoring gut microbiota homeostasis to treat AS.

The heatmap in Figure 7C depicts the composition at the Phylum level, with *Firmicutes*, *Bacteroidota*, *Desulfobacterota*, *Proteobacteria*, and *Actinobacteriota* being the dominant phyla, accounting for more than 97% of the total microbiota. Subsequently, differential gut microbiota from Phylum to Genus levels were screened. Compared to the model group, GXQW treatment enhanced *Bacteroidota* and decreased *Firmicutes* (*P* < 0.05) (Figures 7D,E). At the class level, a lower taxonomic rank of the phylum, GXQW treatment restored *Bacilli* within *Firmicutes*, followed by a sequential restoration of *Erysipelotrichales* within *Bacilli* at the order level, *Erysipelotrichaceae* within *Erysiplotrichales* at the family level, and *Dubosiella* within *Erysipelotrichaceae* at the genus level (Figures 7F–I). Additionally, compared to the control group, the model group presented a higher *Firmicutes*/*Bacteroidota* (F/B) ratio (*P* < 0.05), while this value was lower in the GXQW group than the model group (*P* < 0.05) (Figure 7J).

### 3.6 GXQW alters serum metabolite profiles and identifies key biomarkers in atherosclerotic mice

**FIGURE 8:**
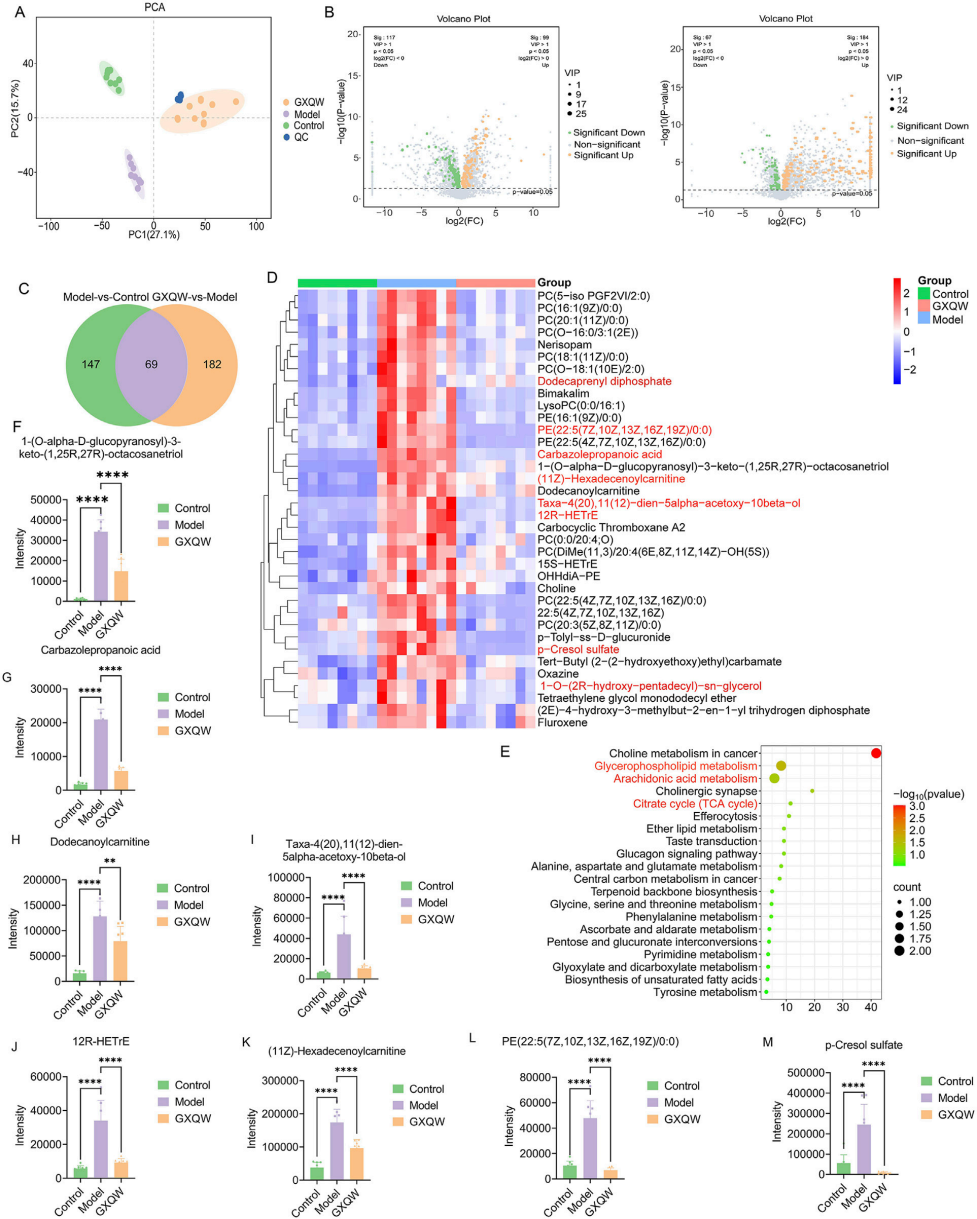
Effects of GXQW on the serum metabolomics of high-fat diet-induced AS mice. **(A)** PCA score plot. **(B)** Volcano plot of the model group vs. control group (left) and GXQW group vs. model group (right). **(C)** Venn diagram of differential metabolites. **(D)** KEGG pathway enrichment of differential metabolites. **(E)** Heatmap of 36 reprogrammed differential metabolites. **(F–M)** Relative abundance statistics of eight potential serum differential metabolite biomarkers. Data are presented as mean ± standard deviation, n = 8. **P < 0.01 and ****P < 0.0001.

In order to investigate the changes in endogenous metabolites in the serum of ApoE^−/−^ mice treated with GXQW, non-targeted metabolomics analysis was performed on their serum. The results are shown in Figure 8. The PCA score plot demonstrated that QC samples clustered tightly together, indicating stable analytical conditions and good reproducibility of the detection process. Furthermore, PCA revealed a clear separation of metabolites between the control, model, and GXQW groups (Figure 8A).

Using the criteria VIP > 1 and *P* < 0.05, 216 differential serum metabolites were identified between the control and model groups. Similarly, 251 differential metabolites were found between the model and GXQW groups (Figure 8B). Among these, 69 metabolites were different in both comparisons (Figure 8C), with 36 metabolites elevated in the high-fat diet-fed mice and downregulated by GXQW treatment (Figure 8D). These 69 differential serum metabolites further underwent KEGG pathway enrichment analysis. The top twenty pathways ranked by count value included Glycerophospholipid metabolism, Citric acid cycle (TCA cycle), and Arachidonic acid metabolism (Figure 8E).

Based on Log_2_FoldChange > 2, eight differential serum metabolic biomarkers were screened, including 1-(O-alpha-D-glucopyranosyl)-3-keto-(1,25R,27R)-octacosanetriol, carbazolepropanoic acid, dodecanoylcarnitine, taxa-4(20),11(12)-dien-5alpha-acetoxy-10beta-ol, 12R-HETrE, (11Z)-hexadecenoylcarnitine, PE (22:5 (7Z,10Z,13Z,16Z,19Z)/0:0), and p-cresol sulfate (PCS). These biomarkers were significantly promoted in the model group and reduced in the GXQW group (*P* < 0.05) (Figures 8F–M).

### 3.7 Positive correlation between GXQW-Altered serum metabolites and *dubosiella* abundance

**FIGURE 9:**
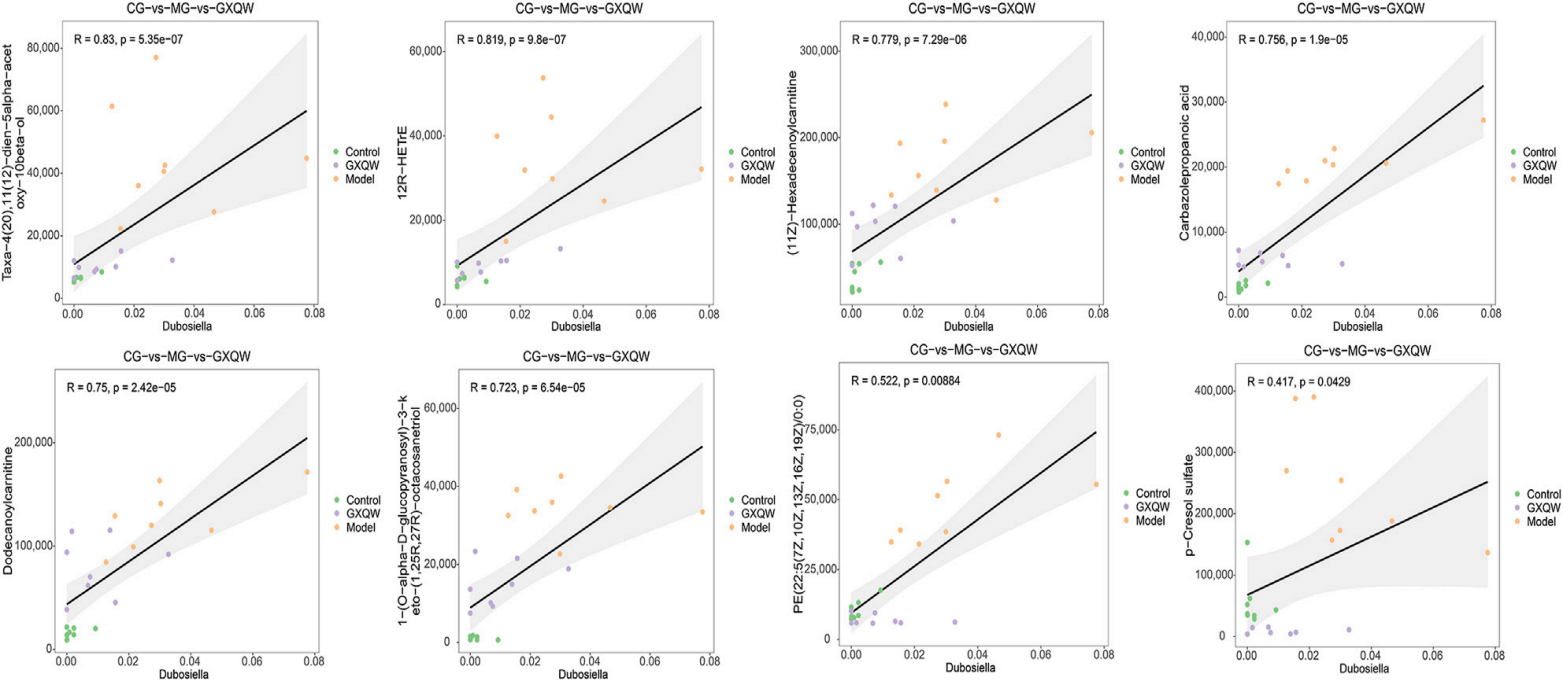
Correlation analysis between *Dubosiella* and eight differential metabolic markers.

In order to explore whether the restoration of gut microbiota dysbiosis by GXQW in ApoE^−/−^ mice was related to changes in serum metabolites, the eight differential serum metabolic markers were analyzed in relation to the characteristic gut microbiota *Dubosiella*. The results are detailed in Figure 9. Results with |R| > 0.06 and *P* < 0.05 were considered to be significantly associated. All eight differential serum metabolites displayed a significant positive correlation with *Dubosiella*.

## 4 Discussion

As is a prevalent and multifactorial cardiovascular disorder that poses a substantial threat to human health. It remains a major contributor to the global burden of cardiovascular diseases, including coronary artery disease and stroke (Gao and Liu, 2017). The rising incidence of AS, driven largely by shifts in lifestyle and dietary patterns, continues to challenge both individual health and public healthcare systems (Islam et al., 2025). Currently, pharmacological intervention is the primary treatment for AS, with surgical procedures and TCM providing supplementary options for specific cases. Additionally, advancements in biomedical technologies, such as nanomedicine, have opened promising new avenues for AS treatment.

TCM plays a crucial role in the prevention and treatment of AS. Its unique pharmacological effects and diverse components effectively improve blood circulation, lower blood lipid levels, and promote the repair and regeneration of the arterial endothelium (Liu et al., 2022; Z et al., 2025; Zhu et al., 2020; Liu et al., 2023). In the GXQW formulation, a considerable number of flavonoid and phenylpropanoid compounds were identified. Flavonoid compounds contribute to the prevention and treatment of AS through mechanisms such as antioxidation, anti-inflammation, antithrombosis, and lipid metabolism regulation (Singh et al., 2024; Lu et al., 2025). Similarly, phenylpropanoid compounds exert anti-AS effects through multiple mechanisms, including the inhibition of foam cell formation, antioxidation, anti-inflammation, and modification of cholesterol metabolism (Cruz Neto et al., 2024; Luque-Sierra et al., 2018; Toma et al., 2020; Yang et al., 2018; Marrelli et al., 2021; Ma et al., 2023). Both flavonoids and phenylpropanoids interact dynamically with the gut microbiota, forming a “compound-microbiota-host” tripartite regulatory network. This mutualistic relationship provides scientific evidence for the development of microbiota-targeted TCM intervention strategies (Fu et al., 2025).

The gut microbiota and serum metabolites are integral in the onset and progression of AS. They not only influence the metabolic state of the body but also impact vascular health through intricate interactions (Zhao et al., 2022). Studies have shown that the microbial communities in the gut can modulate the host’s immune response and inflammation levels by producing various metabolites, thus contributing to the pathogenesis of AS. Meanwhile, serum metabolites serve as dynamic biomarkers of systemic metabolic status and significantly impact vascular cell function and lipid homeostasis (Nie et al., 2019). The results of this study demonstrate significant changes in the composition of the gut microbiota in mice, with a reduction in the relative abundance of the F/B ratio. This is consistent with previous reports on the relationship between obesity and the F/B ratio (Qi et al., 2024; Liang et al., 2024; Lee et al., 2024). Moreover, GXQW treatment significantly reduced the abundance of *Dubosiella*. This genus is a relatively new and increasingly recognized bacterium in the gut microbiota, with research indicating its close relationship with the host’s metabolic health, particularly in hepatic function and lipid metabolism (Han et al., 2024; Xu et al., 2023; Zhang et al., 2021; Huan et al., 2025; Yang et al., 2023; Liu W. et al., 2024; Xiao et al., 2023). Specifically, (1) the FXR-FGF15/19 signaling pathway: *Dubosiella* modulates gut bile acid composition (e.g., DCA, CA) to activate FXR and inhibit hepatic CYP7A1, thereby regulating cholesterol metabolism. (2) The TLR4/NF-κB inflammatory signaling pathway: *Dubosiella* enhances the intestinal barrier, promotes the translocation of LPS into the bloodstream, and activates TLR4, leading to hepatic inflammation and lipid dysregulation.

Dysbiosis of the gut microbiota is a significant contributor to vascular inflammation, with prolonged dysbiosis eliciting immune responses and leading to a chronic inflammatory state (Piccioni et al., 2021). Moreover, systemic inflammation mediated by the gut microbiota is closely associated with the pathophysiological mechanisms underlying obesity-related AS (Luqman et al., 2024). This suggests that microbial dysbiosis facilitates the abnormal accumulation of pro-atherogenic metabolites, thereby activating platelets and inducing a persistent hyperemic response within the body (Islam et al., 2025). In this study, GXQW effectively regulated the abundance of key bacterial phyla implicated in AS progression, such as *Bacteroidota* and *Firmicutes* (Gan et al., 2024; Liu et al., 2025).

The untargeted metabolomics results identified significant enrichment in three pivotal metabolic pathways: Glycerophospholipid metabolism, TCA cycle, and Arachidonic acid metabolism. These pathways are critically involved in the pathogenesis of AS (Sun-Waterhouse et al., 2024; Zhu et al., 2016): 1. Glycerophospholipid metabolism: Phospholipids constitute a fundamental component of AS plaques. In particular, oxidized phospholipids (oxPLs) can promote endothelial cell damage, macrophage inflammatory activation, and foam cell formation. LysoPC (lysophosphatidylcholine), a pivotal intermediate in glycerophospholipid metabolism, can enhance monocyte adhesion and migration and trigger inflammatory responses. 2. TCA cycle: The TCA cycle is a central pathway in energy metabolism, closely linked to macrophage polarization, ROS generation, and lipid oxidation. In AS animal models, mitochondrial dysfunction or disruption of the TCA cycle can promote ROS production, induce endothelial cell apoptosis, and lead to the accumulation of succinate during M1 macrophage activation, thus exacerbating inflammation. 3. Arachidonic acid metabolism: Metabolites of arachidonic acid (AA), including prostaglandins (PGs), leukotrienes (LTs), and thromboxanes (TXs), are potent molecules involved in inflammation and vascular regulation. Aberrant AA metabolism can lead to vasoconstriction, platelet aggregation, and endothelial injury, accelerating plaque development.

In this study, GXQW reversed the elevation of eight metabolites in the serum of high-fat diet-induced ApoE^−/−^ mice, including 1-(O-alpha-D-glucopyranosyl)-3-keto-(1,25R,27R)-octacosanetriol, Carbazolepropanoic acid, Dodecanoylcarnitine, Taxa-4(20),11(12)-dien-5alpha-acetoxy-10beta-ol, 12R-HETrE, (11Z)-Hexadecenoylcarnitine, PE (22:5 (7Z,10Z,13Z,16Z,19Z)/0:0), and PCS. Previous studies have demonstrated that Dodecanoylcarnitine, 12R-HETrE, (11Z)-Hexadecenoylcarnitine, PE (22:5 (7Z,10Z,13Z,16Z,19Z)/0:0), and PCS play key roles in the pathogenesis and progression of AS. Dodecanoylcarnitine stimulates endothelial cells to secrete inflammatory factors, such as interleukin-6 (IL-6) and tumor necrosis factor-alpha (TNF-α), thereby promoting plaque formation and progression (Zhang et al., 2025). 12R-HETrE enhances endothelial permeability, facilitating lipid infiltration into the subendothelial space. Additionally, it promotes the chemotaxis of neutrophils, attracting inflammatory cells to the blood vessel wall, triggering an inflammatory response, further damaging the endothelial cells, and accelerating plaque formation in AS (du Toit et al., 2023). (11Z)-Hexadecenoylcarnitine directly participates in the β-oxidation of fatty acids and contributes to ROS accumulation. ROS damages endothelial cells, impairing endothelial function, promoting the adhesion and migration of inflammatory cells and cytokine release, and accelerating the progression of AS (Hao et al., 2024). Following phospholipid hydrolysis, PE (22:5 (7Z,10Z,13Z,16Z,19Z)/0:0) releases a large amount of arachidonic acid, which further generates inflammatory mediators such as prostaglandins and leukotrienes, accumulating immune cells and promoting inflammation and plaque development. Meanwhile, PE (22:5 (7Z,10Z,13Z,16Z,19Z)/0:0) is oxidized into oxidized oxPLs, increasing endothelial permeability and enhancing the generation of ox-LDL, which is taken up by macrophages and forms foam cells, marking the early stages of AS (Chen et al., 2024; Keller et al., 2022). PCS, a uremic toxin derived from gut microbiota metabolism, induces oxidative stress in endothelial cells and activates the NF-κB pathway, leading to increased expression and release of inflammatory cytokines like IL-6 and TNF-α, further promoting the onset and progression of AS (Kato et al., 2021; Cao et al., 2024).

In this study, the role of *Dubosiella* and PCS is particularly prominent, because the metabolic activity of *Dubosiella* can produce a variety of sulfur-containing compounds and phenolic metabolites, such as p-cresol is one of its important metabolites. p-Cresol is produced in the liver by sulfatase to PCS, a protein-binding uremic toxin with low water solubility and long half-life, which accumulates in the blood. GXQW may inhibit intrusive atherosclerosis by modulating the *Dubosiella*-PCS axis because PCS activates NADPH oxidase, leading to increased production of reactive oxygen species (ROS) in vascular endothelial cells, leading to oxidative damage (Wu et al., 2021). At the same time, ROS can activate the NF-κB pathway, induce the expression of adhesion molecules (e.g., VCAM-1, ICAM-1) and pro-inflammatory cytokines (e.g., IL-6, TNF-α), promote the adhesion of monocytes to the vascular endothelium, and initiate early atherosclerotic lesions. PCS may disrupt endothelial cell tight junction proteins (e.g., ZO-1, VE-cadherin), resulting in increased vascular permeability and lipid and inflammatory cell infiltration into the subendothelial space, resulting in lipid deposition. PCS upregulates the expression of macrophage scavenger receptors (e.g., CD36, SR-A), enhances their uptake of oxidized low-density lipoprotein (oxLDL), and accelerates foam cell formation, a key step in the formation of lipid cores in atherosclerotic plaques. PCS may inhibit ATP-binding cassette transporter A1 (ABCA1)-mediated transport of cholesterol from macrophages to high-density lipoprotein (HDL), resulting in cholesterol accumulation within the blood vessel wall (Lin et al., 2015). PCS induces the transition of VSMCs from contractile (high expression of α-SMA) to synthetic (low expression of α-SMA, high expression of proliferation-related proteins), enhances their migration capacity, migrates to the intimal layer and secretes extracellular matrix, and promotes plaque cap formation and vascular remodeling. PCS may promote VSMC proliferation by activating MAPK (e.g., ERK1/2) or PI3K/Akt pathways, resulting in thickening of the vessel wall and increased plaque volume (Tsai et al., 2015). The results in this study show that GXQW can well modulate *Dubosiella* and PCS, providing a theoretical basis for GXQW to fight atherosclerosis.

This study demonstrated that GXQW had certain effects in regulating gut microbiota and serum metabolites. However, the study is subject to several limitations. First, 16S rRNA gene sequencing has a detection limit and can only identify genera. It is difficult to precisely distinguish between closely related species. This may lead to the omission of low-abundance metabolites and result in an inaccurate microbiota structure analysis, potentially masking the subtle correlations between specific microbial species and metabolic changes (Zhang et al., 2024; Fuks et al., 2018; Liu Y. F. et al., 2024). Second, due to constraints from manpower, resources, and time, the study was conducted exclusively in a murine model without validation in human subjects. Given the considerable inter-individual variability in gut microbiota composition and metabolic phenotypes across human populations, these findings may lack generalizability and carry a risk of spurious associations. Finally, while correlations between gut microbiota and serum metabolites were observed, the causal direction of these associations remains unclear—whether specific microbiota changes drive metabolic alterations or *vice versa* warrants further mechanistic investigation.

In our study, a high dosage of 1.8 g/kg of the extract was employed to ensure measurable pharmacological effects in the experimental model. However, it is important to recognize that such an elevated dose may introduce potential artifacts, including off-target effects or physiological responses unrelated to the intended therapeutic mechanisms. These confounding factors may complicate data interpretation and reduce the translational relevance of the findings to clinical settings, where lower and more precisely defined dosages are typically necessary. To overcome this limitation, future research will focus on optimizing the extract preparation using efficient cooling technology. This advanced processing technique is expected to improve the bioavailability and potency of the active constituents, thereby allowing for a reduction in the required therapeutic dose. By refining both extraction and formulation procedures, we aim to achieve equivalent or even enhanced pharmacodynamic outcomes at substantially lower dosages. Such optimization would not only reduce the risk of dose-related artifacts but also enhance the safety profile and clinical applicability of the extract. Moreover, dose reduction through process improvement will enable a more accurate elucidation of the extract’s mechanisms of action by minimizing background noise and unintended biological interference. This will facilitate a clearer understanding of its therapeutic potential, especially in complex disease models where excessive dosing may provoke compensatory or stress responses unrelated to the primary therapeutic targets.

Research on GXQW has predominantly focused on animal experiments, lacking clinical investigations. Future efforts should prioritize the implementation of large-scale, multi-center, randomized, double-blind, placebo-controlled clinical trials to rigorously assess the efficacy and safety of GXQW in human populations. Given the regional variability in gut microbiota composition and the epidemiological patterns of AS, tailored clinical trial designs are essential to evaluate the therapeutic effects of GXQW across diverse demographic groups. Furthermore, combining GXQW with standard-of-care pharmacotherapies, such as statins, may help optimize integrative treatment regimens, providing more effective strategies for clinical AS treatment.

## 5 Conclusion

**FIGURE 10:**
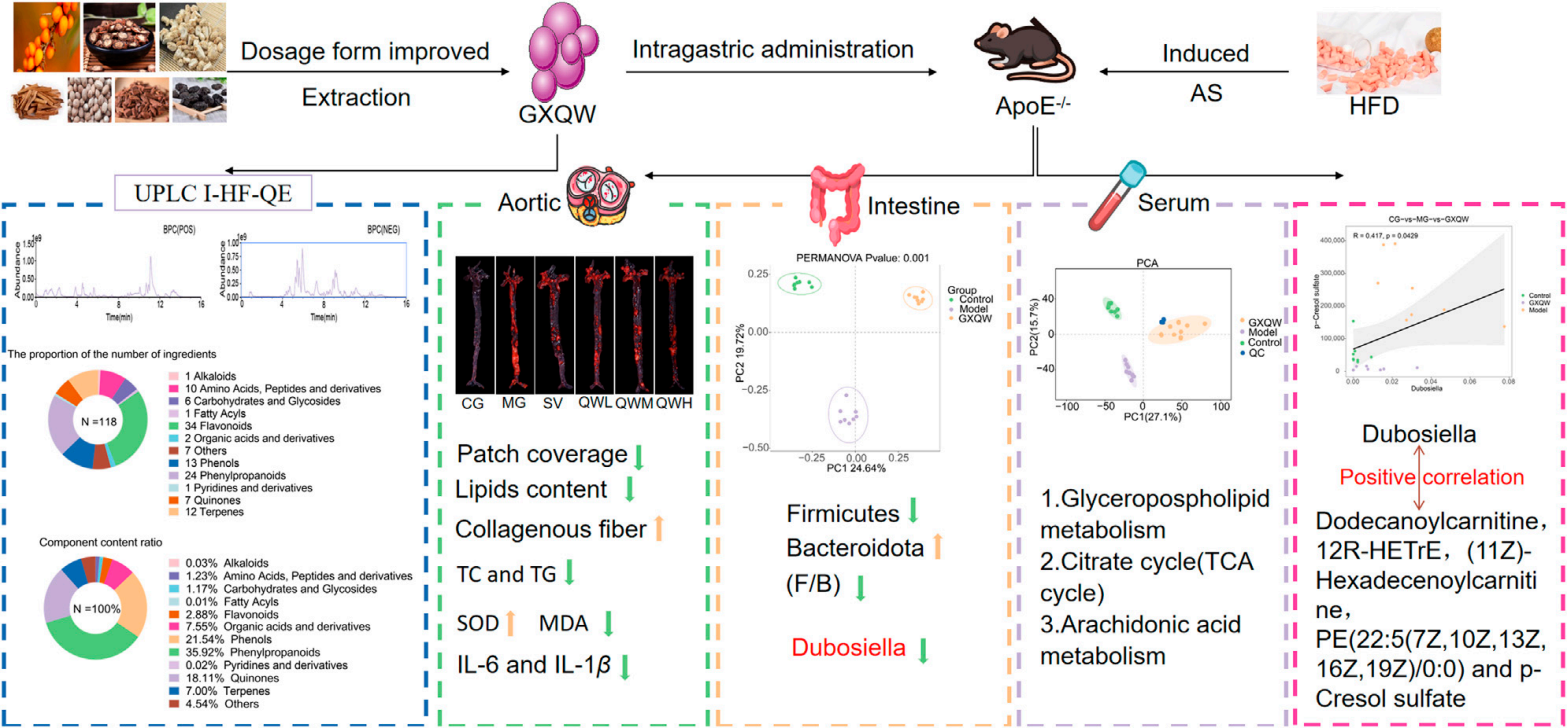
GXQW alleviates high-fat diet-induced AS in mice by improving intestinal flora dysbiosis and serum metabolic disorder.

In summary, GXQW demonstrates significant therapeutic potential in alleviating AS-related pathological features. Mechanistically, GXQW modulates the abundance of *Dubosiella* and regulates key serum metabolites, including 1-(O-alpha-D-glucopyranosyl)-3-keto-(1,25R,27R)-octacosanetriol, Carbazolepropanoic acid, Dodecanoylcarnitine, Taxa-4(20),11(12)-dien-5alpha-acetoxy-10beta-ol, 12R-HETrE, (11Z)-Hexadecenoylcarnitine, PE (22:5 (7Z,10Z,13Z,16Z,19Z)/0:0), and PCS. Additionally, GXQW influences several critical metabolic pathways implicated in lipid metabolism and inflammation, including Glycerophospholipid metabolism (Figure 10), TCA cycle, and Arachidonic acid metabolism. These findings provide compelling preclinical evidence supporting the clinical utility of GXQW and the potential of TCM in regulating gut microbiota to improve AS.

## Data Availability

The 16S sequence dataset generated in this study has been deposited in the NCBI Sequence Read Archive (SRA) database (PRJNA1276683). All data generated during this study are included in the article/Supplementary Material.
