# Supplementary material for: The mechanism of Guanxin Qiwei dropping pills target Dubosiella to improve atherosclerosis
Source: Front Pharmacol. 2025 Jul 1;16:1633862. doi: 10.3389/fphar.2025.1633862 (PMC12260407; doi:10.3389/fphar.2025.1633862)
Supplement: Supplementary file 1 [file Table1.docx]

Supplementary Material

Table S1: Experimental Reagents and Consumables

| **Name** | **Model number** | **Manufacturer** |
| --- | --- | --- |
| High fat feed | D12079B | Beijing Keao Xieli Feed Co.,Ltd. |
| mouse IL-1βELISA reagent kit | KTE7005 | Abbkine Biotechnology Co., Ltd |
| mouse IL-6 ELISA reagent kit | KTE7009 | Abbkine Biotechnology Co., Ltd |
| Cholesterol (TC) reagent kit | KTB2220 | Abbkine Biotechnology Co., Ltd |
| Triglyceride (TG) test kit | KTB2200 | Abbkine Biotechnology Co., Ltd |
| High density lipoprotein (HDL) test kit | KTB2250 | Abbkine Biotechnology Co., Ltd |
| Low density lipoprotein (LDL) test kit | KTB2260 | Abbkine Biotechnology Co., Ltd |
| Lipid peroxide malondialdehyde(MDA) | KTB1050 | Abbkine Biotechnology Co., Ltd |
| Superoxide dismutase（SOD） | KTB1030 | Abbkine Biotechnology Co., Ltd |
| 4% polyformaldehyde | G1101 | Wuhan Servicebio Technology CO.,LTD |
| Hematoxylin | G1004 | Wuhan Servicebio Technology CO.,LTD |
| Eosin Staining Solution | G1001 | Wuhan Servicebio Technology CO.,LTD |
| Masson's Trichrome Staining Kit | G1340 | Beijing Solarbio Science & Technology Co., Ltd |
| OilRed O Solution | G2013 | Wuhan Servicebio Technology CO.,LTD |
| Optimal cutting temperature compound | G6059 | Wuhan Servicebio Technology CO.,LTD |
| Simvastatin Tablets | 230104 | Shandong Lukang Group Saite Co., Ltd |
| Sodium carboxymethyl cellulose | 419273 | Sigma-Aldrich |

TableS2 GXQW prescription ratio and drug information

| **Chinese medicine name** | **Latin name** | **Medical plants name** | **Parts used** | **Batch number** | **source** |
| --- | --- | --- | --- | --- | --- |
| Roudoukou | *Myristicae semen* | *Myristica fragrans* Houtt. | Dried seed kernels | 220101-02 | Chengdu Qilong Traditional Chinese Medicine Slices Co., LTD (Sichuan, China) |
| Guangzao | *Choerospondiatis fructus* | *Choerospondias* *axillaris* (Roxb.) B.L.Burtt & A.W.Hill | Dried fruits | 170901 | Bozhou Qiaocheng district all things Xiang Chinese Medicine slices Co., LTD (Anhui,China) |
| Tanxiang | *Santali albi lignum* | *Santalum album* L. | Dried heartwood | 221001 | Sichuan Hongkangyuan Pharmaceutical Co., LTD.(Sichuan,China) |
| Danshen | *Codonopisis radix* | *Codonopsis pilosula* (Franch.) Nannf. | Dried roots | 230601 | Anhui Yaozhiyuan Chinese herbal Medicine Co.,LTD (Anhui,China) |
| Shannai | *Kaempferiae Rhizoma* | *Kaempferia galanga* L. | Dried rhizomes | A220208 | Bozhou Yonggang Decoction pieces Factory Co., LTD  (Anhui,China) |
| Jiangxiang | *Dalbergiae odoriferae lignum* | *Dalbergia odorifera* T.C.Chen | Dried heartwood | 210401 | Anhui Yaozhiyuan Chinese herbal Medicine Co.,LTD (Anhui,China) |
| Shaji | *Hippophae Fructus* | *Hippophae rhamnoides* L. | Dried fruits | 710220901 | Anguo city Yuanguang Pharmaceutical Co., LTD  （Hebe, China) |

Table S3 Elution gradient information table

| **Times** | **A%** | **B%** | **Times** | **A%** | **B%** |
| --- | --- | --- | --- | --- | --- |
| 0 | 95 | 5 | 14 | 0 | 100 |
| 2 | 95 | 5 | 15 | 0 | 100 |
| 4 | 70 | 30 | 15.1 | 95 | 5 |
| 8 | 50 | 50 | 16 | 95 | 5 |
| 10 | 20 | 80 |  |  |  |

Table S4 Mass spectrometry parameter information table

| **Argument** | **(+)** | **(-)** |
| --- | --- | --- |
| Spray Voltage (V) | 3800 | -3000 |
| Capillary Temperature (°C) | 320 | 320 |
| Aux gas heater temperature (℃) | 350 | 350 |
| Sheath Gas Flow Rate (Arb) | 35 | 35 |
| Aux gas flow rate (Arb) | 8 | 8 |
| S-lens RF level | 50 | 50 |
| Mass range (*m/z*) | 100-1200 | 100-1200 |
| Full ms resolution | 70000 | 70000 |
| MS/MS resolution | 17500 | 17500 |
| NCE/stepped NCE | 10，20，40 | 10，20，40 |

Table S5 Elution gradient

| **time** | **A%** | **B%** |
| --- | --- | --- |
| 0.0 | 95.0 | 5.0 |
| 2.0 | 95.0 | 5.0 |
| 4.0 | 70.0 | 30.0 |
| 8.0 | 50.0 | 50.0 |
| 10.0 | 20.0 | 80.0 |
| 14.0 | 0.0 | 100.0 |
| 15.0 | 0.0 | 100.0 |
| 15.1 | 95.0 | 5.0 |
| 16.0 | 95.0 | 5.0 |

Table S6 Mass spectrum parameters

| **argument** | **Positive ion** | **Negative ion** |
| --- | --- | --- |
| Spray Voltage (V) | 3800 | -3000 |
| Capillary Temperature (°C) | 320 | 320 |
| Aux gas heater temperature (℃) | 350 | 350 |
| Sheath Gas Flow Rate (Arb) | 35 | 35 |
| Aux gas flow rate (Arb) | 8 | 8 |
| S-lens RF level | 50 | 50 |
| Mass range (*m/z*) | 70-1050 | 70-1050 |
| Full ms resolution | 70000 | 70000 |
| MS/MS resolution | 17500 | 17500 |
| NCE/stepped NCE | 10,20,40 | 10,20,40 |

Table S7 GXQW in vitro ingredients

| No | Metabolites | Formula | Adducts | theoretical m/z | m/z | Retention time (min) | Mass Error (ppm) | Ion mode | Fragment Ions | sort |
| --- | --- | --- | --- | --- | --- | --- | --- | --- | --- | --- |
| 1 | L-Pipecolic acid^[1-5]^ | C_6_H_11_NO_2_ | [M+H]^+^ | 130.0863 | 130.0862 | 0.75 | -0.43 | POS | 84.0443,84.0807,130.086 | B |
| 2 | L-Lysine^[1-5]^ | C_6_H_14_N_2_O_2_ | [M+H-H_2_O]^+^,[M+H]^+^ | 147.1128 | 147.1126 | 0.75 | -1.16 | POS | 84.0807,130.086 | B |
| 3 | L-Histidine^[1-5]^ | C_6_H_9_N_3_O_2_ | [M+H]^+^ | 156.0768 | 156.0766 | 0.77 | -0.89 | POS | 95.0602,110.0711,156.0764 | B |
| 4 | L-Arginine^[1-5]^ | C_6_H_14_N_4_O_2_ | [M+H]^+^,[M+Na]^+^ | 175.119 | 175.1188 | 0.78 | -0.91 | POS | 60.0557,70.0651,84.0807,112.0758,116.0704,130.0865,130.0971,158.092,175.1186 | B |
| 5 | Mannosamine^[1-5]^ | C_6_H_13_NO_5_ | [M+H-H_2_O]^+^,[M+H]^+^ | 162.0761 | 162.0761 | 0.85 | -0.18 | POS | 102.0546,102.9703,114.0548,115.0388,126.0548,127.0387,134.0681,143.9972,144.0653,162.0757 | C |
| 6 | 6-(alpha-D-Glucosaminyl)-1D-myo-inositol^[2,3]^ | C_12_H_23_NO_10_ | [M+H]^+^ | 342.1395 | 342.139 | 0.87 | -1.27 | POS | 145.0493,162.0758,163.0596,174.0756,240.0865,288.1067,306.1172,324.1278,325.1156,342.1383 | C |
| 7 | Trehalose^[1,2]^ | C_12_H_22_O_11_ | [M+H]^+^,[[M+NH4]+]^+^,[M+Na]^+^,[M+H-H_2_O]^+^ | 365.1055 | 365.1045 | 0.9 | -2.79 | POS | 127.0388,145.0493,163.0597,180.0864,198.0967,234.0652,264.0757,264.5773,289.0914,325.1122 | C |
| 8 | Cyclic N-Acetyl-D-mannosamine^[3]^ | C_8_H_15_NO_6_ | [M+Na]^+^ | 244.0792 | 244.0789 | 0.92 | -0.97 | POS | 112.0505,185.9594,186.9114,203.0524,244.0788 | C |
| 9 | L-Threonine^[3]^ | C_4_H_9_NO_3_ | [M+H]^+^ | 120.0655 | 120.0654 | 0.99 | -0.96 | POS | 56.0496,74.06,102.0548,120.0654 | B |
| 12 | Chebulic acid^[3]^ | C_14_H_12_O_11_ | [M-H]^-^ | 355.0307 | 355.0297 | 1.17 | -2.68 | NEG | 92.927,94.9241,112.9845,162.8387,167.0202,176.8999,296.8119,337.0196,355.0296 | H |
| 13 | Nicotinic acid riboside^[3]^ | C_11_H_13_NO_6_ | [M+H]^+^ | 256.0816 | 256.0813 | 1.18 | -1.06 | POS | 109.0282,114.0548,127.0387,130.0496,142.0497,192.0651,215.0159,226.0706,238.0704,256.0809 | A |
| 14 | 3-Hydroxypicolinic acid^[4-8]^ | C_6_H_5_NO_3_ | [M+H]^+^ | 140.0342 | 140.0342 | 1.18 | -0.22 | POS | 112.0392,140.034 | J |
| 15 | Citric acid^[4-8]^ | C_6_H_8_O_7_ | [M-H]^-^ | 191.0197 | 191.0189 | 1.19 | -4.19 | NEG | 85.0283,87.0076,111.0077,129.0182,173.0081,191.0191 | F |
| 16 | 2-Methylcitric acid^[4-8]^ | C_7_H_10_O_7_ | [M-H_2_O-H]^-^ | 187.0247 | 187.024 | 1.33 | -4.18 | NEG | 99.0438,115.0385,125.0231,125.0958,143.0337,187.024 | F |
| 17 | L-Leucine^[4-8]^ | C_6_H_13_NO_2_ | [M+H]^+^ | 132.1019 | 132.1017 | 1.52 | -1.51 | POS | 86.0963,90.0549,132.0764 | B |
| 18 | N-(1-Deoxy-1-fructosyl)phenylalanine^[8,9]^ | C_15_H_21_NO_7_ | [M+H-H_2_O]^+^,[M+H]^+^ | 328.1391 | 328.138 | 2.33 | -3.39 | POS | 120.0803,132.0806,166.0858,178.0857,182.081,264.1229,274.0916,292.1175,310.1277 | B |
| 19 | 5-Hydroxymethylfurfural^[8,9]^ | C_6_H_6_O_3_ | [M+H-H_2_O]^+^ | 109.0284 | 109.0286 | 2.57 | 1.35 | POS |  | G |
| 20 | Pantothenic acid^[8,9]^ | C_9_H_17_NO_5_ | [M+H]^+^,[M+Na]^+^ | 220.118 | 220.1174 | 2.77 | -2.68 | POS | 72.0441,85.0644,90.0549,103.0753,116.0342,124.0756,184.0969,202.1071,205.1583,220.1174 | B |
| 21 | Androsin^[8,9]^ | C_15_H_20_O_8_ | [M-H]^-^ | 327.1085 | 327.1078 | 4.05 | -2.31 | NEG | 102.947,113.0227,116.9271,146.9377,151.0382,165.0545,190.9277,210.5938,327.1086 | C |
| 22 | Cis-Ferulic acid 4-O-beta-D-glucopyranoside^[8,9]^ | C_16_H_20_O_9_ | [M+FA-H]^-^ | 401.109 | 401.1082 | 4.09 | -2.15 | NEG | 129.9744,134.0365,149.0591,193.0494,197.0418,209.4067,210.2433,266.0183,355.1039,401.1028 | C |
| 23 | Chlorogenic acid^[8,9]^ | C_16_H_18_O_9_ | [M-H]^-^,[M+FA-H]^-^ | 399.0934 | 399.0926 | 4.22 | -1.97 | NEG | 144.9912,146.9377,174.8772,190.9281,191.0555,212.0019,236.8775,280.8679,304.9701,353.0882 | I |
| 24 | Kaempferol 3-sophoroside-7-rhamnoside^[8,9]^ | C_33_H_40_O_20_ | [M-H]^-^,[M+FA-H]^-^ | 801.2095 | 801.2101 | 4.3 | 0.76 | NEG |  | E |
| 25 | Isorhamnetin 3-sophoroside-7-rhamnoside^[10]^ | C_34_H_42_O_21_ | [M-H]^-^,[M+FA-H]^-^ | 831.22 | 831.2203 | 4.36 | 0.33 | NEG | 299.0202,313.0351,314.0427,623.1623,639.1559,785.2148 | E |
| 26 | Fraxin^[1]^ | C_16_H_18_O_10_ | [M-H]^-^ | 369.0827 | 369.082 | 4.4 | -2.02 | NEG | 85.0284,113.0232,175.0237,178.026,193.0499,207.029,369.0818 | I |
| 27 | Esculetin^[1]^ | C_9_H_6_O_4_ | [M+H]^+^ | 179.0339 | 179.034 | 4.45 | 0.55 | POS | 73.0285,89.0599,105.0336,133.0651,161.0595,179.0338 | I |
| 28 | Leu-Leu^[1]^ | C_12_H_24_N_2_O_3_ | [M+H]^+^ | 245.186 | 245.1857 | 4.49 | -1.16 | POS |  | B |
| 29 | 2"-O-beta-L-galactopyranosylorientin^[1]^ | C_27_H_30_O_16_ | [M+H]^+^ | 611.1607 | 611.1608 | 4.5 | 0.3 | POS | 303.0491,449.1071,611.1573 | E |
| 31 | Quercetin 3-o-neohesperidoside^[1]^ | C_27_H_30_O_16_ | [M-H]^-^ | 609.1461 | 609.1455 | 4.52 | -0.95 | NEG | 299.0194,301.0341,446.0836,609.1471 | E |
| 30 | salvianic acid c^[1]^ | C_18_H_18_O_9_ | [M-H]^-^,2[M-H]^-^ | 377.0878 | 377.0871 | 4.52 | -1.96 | NEG | 135.0436,137.0227,161.0232,179.0335,197.0443,359.0764,377.0882 | G |
| 32 | 6-Hydroxykaempferol 3,6-diglucoside^[1,2]^ | C_27_H_30_O_17_ | [M-H]^-^ | 625.141 | 625.1404 | 4.58 | -0.99 | NEG | 208.5806,300.0286,316.0226,625.1406 | E |
| 33 | Isovanillin^[1]^ | C_8_H_8_O_3_ | [M+H]^+^ | 153.0546 | 153.0544 | 4.71 | -1.14 | POS | 94.0412,109.0646,135.0437,153.0542 | H |
| 34 | Fraxetin^[1]^ | C_10_H_8_O_5_ | [M-H]^-^ | 207.0299 | 207.0292 | 4.71 | -3.14 | NEG |  | I |
| 35 | Quercetin-3-O-D-glucosyl]-(1-2)-L-rhamnoside^[1]^ | C_27_H_30_O_16_ | [M-H]^-^ | 609.1461 | 609.1454 | 4.77 | -1.22 | NEG | 300.0271,609.1459 | E |
| 36 | Quercetin 3-o-(6''-galloyl)-beta-d-glucopyranoside^[1]^ | C_28_H_24_O_16_ | [M-H]^-^ | 615.0992 | 615.099 | 4.77 | -0.32 | NEG |  | E |
| 37 | Rutin^[1]^ | C_27_H_30_O_16_ | [M+H]^+^ | 611.1607 | 611.1601 | 4.78 | -0.87 | POS |  | E |
| 38 | Genipin^[1,2]^ | C_11_H_14_O_5_ | [M-H]^-^ | 225.0768 | 225.0762 | 4.82 | -2.99 | NEG |  | L |
| 39 | Isoquercitrin^[1,2]^ | C_21_H_20_O_12_ | [M+FA-H]^-^ | 509.0938 | 509.0924 | 4.88 | -2.76 | NEG |  | E |
| 40 | Ethyl gallate^[1,2]^ | C_9_H_10_O_5_ | [M+H-H_2_O]^+^ | 181.0495 | 181.0495 | 4.89 | 0.07 | POS |  | H |
| 41 | Ellagic acid^[1,2]^ | C_14_H_6_O_8_ | [M-H]^-^,2[M-H]^-^ | 300.999 | 300.9983 | 4.92 | -2.21 | NEG | 300.9983 | H |
| 42 | 3,3'-Di-O-methylellagic acid 4'-glucoside^[1,2]^ | C_22_H_20_O_13_ | [M-H]^-^ | 491.0831 | 491.0821 | 4.96 | -2.09 | NEG | 312.9985,328.0221,491.0829 | I |
| 43 | Salvianolic acid E^[1,2]^ | C_36_H_30_O_16_ | [M+H]^+^ | 719.1607 | 719.1603 | 5.04 | -0.49 | POS |  | I |
| 44 | Quercitrin^[1,2]^ | C_21_H_20_O_11_ | [M-H]^-^ | 447.0933 | 447.0929 | 5.21 | -0.84 | NEG | 207.9746,208.1117,208.4686,271.0606,299.9933,300.0259,301.0348,315.0138,447.0842,447.0985 | E |
| 45 | Iristectorin B^[1,2]^ | C_23_H_24_O_12_ | [M-H]^-^ | 491.1195 | 491.1183 | 5.21 | -2.54 | NEG | 285.0403,300.0286,300.0593,315.0494,315.0870,315.1248,393.5110,411.1992,428.1989,491.1203 | E |
| 46 | Methylsyringin^[1,2]^ | C_18_H_26_O_9_ | [M-H]^-^ | 385.1504 | 385.1499 | 5.27 | -1.37 | NEG | 85.0283,113.0232,175.0236,209.1171,322.9492,385.151 | L |
| 47 | (2R)-3-(3,4-dihydroxyphenyl)-2-[(Z)-3-(3,4-dihydroxyphenyl)acryloyl]oxy-propionic acid^[3-10]^ | C_18_H_16_O_8_ | [M-H]^-^,[M+FA-H]^-^ | 359.0772 | 359.0763 | 5.29 | -2.62 | NEG | 72.9918,135.0439,161.0233,179.0338,197.0446,359.0766 | G |
| 48 | Lithospermic acid^[1,2]^ | C_27_H_22_O_12_ | [M-H_2_O-H]^-^ | 519.0932 | 519.0921 | 5.41 | -2.21 | NEG | 185.0233,277.0498,279.0294,293.045,295.0604,321.0398,339.05,519.0926 | H |
| 49 | Salvianolic acid Y^[1,2]^ | C_36_H_30_O_16_ | [M+H-H_2_O]^+^ | 701.1501 | 701.1503 | 5.42 | 0.27 | POS |  | I |
| 50 | Propyl gallate^[1,2]^ | C_10_H_12_O_5_ | [M-H-H_2_O]^-^ | 193.0505 | 193.0497 | 5.53 | -4.5 | NEG | 108.0204,134.0359,149.0596,163.0391,178.0625,192.9895,193.0496 | H |
| 51 | salvianolic acid n^[1,2]^ | C_26_H_22_O_10_ | [M-H]^-^ | 493.114 | 493.1134 | 5.73 | -1.31 | NEG | 109.0279,185.0232,197.0442,295.0607,313.0702,493.113 | G |
| 52 | Salvianolic acid A^[1,2]^ | C_26_H_22_O_10_ | [M+H]^+^,[M+NH_4_]^+^ | 495.1286 | 495.1287 | 5.74 | 0.16 | POS |  | I |
| 53 | Liquiritigenin^[1,2]^ | C_15_H_12_O_4_ | [M+FA-H]^-^ | 301.0719 | 301.0711 | 5.8 | -2.67 | NEG | 177.018,197.0022,217.0087,256.9815,268.0374,283.0604,301.0001,301.0709 | E |
| 54 | Licochalcone B^[9,10]^ | C_16_H_14_O_5_ | [M+FA-H]^-^ | 331.0824 | 331.0816 | 5.8 | -2.57 | NEG |  | E |
| 55 | Tricin^[9,10]^ | C_17_H_14_O_7_ | [M-H_2_O-H]^-^ | 311.0561 | 311.0555 | 5.86 | -1.82 | NEG | 174.9553,207.7107,267.0654,311.0165,311.0549 | E |
| 56 | salvianolic acid j^[9,10]^ | C_27_H_22_O_12_ | [M-H_2_O-H]^-^ | 519.0933 | 519.0923 | 6.14 | -1.79 | NEG | 185.0235,277.0502,279.029,293.0448,295.0604,321.0398,339.0507,519.0928 | G |
| 57 | Quercetin^[9,10]^ | C_15_H_10_O_7_ | [M-H]^-^ | 301.0354 | 301.0347 | 6.16 | -2.1 | NEG | 151.0025,178.9971,197.0026,217.0085,301.0002,301.0353 | E |
| 58 | Ethyl Caffeic acid^[9,10]^ | C_11_H_12_O_4_ | [M-H]^-^ | 207.0663 | 207.0653 | 6.49 | -4.49 | NEG | 135.0441,161.0443,179.0339,186.998,206.9875,207.0654 | I |
| 59 | Echinatin^[9,10]^ | C_16_H_14_O_4_ | [M+FA-H]^-^ | 315.0875 | 315.0869 | 6.71 | -2.04 | NEG | 109.0281,315.0872 | E |
| 60 | Alismoxide^[9,10]^ | C_15_H_26_O_2_ | [M+H-H_2_O]^+^ | 221.19 | 221.1898 | 6.71 | -0.79 | POS | 149.1322,161.1320,163.1477,165.0542,175.1470,203.1061,203.1788,221.1167,221.1547,221.1891 | L |
| 61 | Ferulic acid methyl ester^[9,10]^ | C_11_H_12_O_4_ | [M+H]^+^ | 209.0808 | 209.0807 | 6.72 | -0.58 | POS |  | I |
| 62 | Kajiichigoside F1^[9,10]^ | C_36_H_58_O_10_ | [M+FA-H]^-^ | 695.4012 | 695.401 | 6.93 | -0.31 | NEG |  | L |
| 63 | Pterostilbene^[5]^ | C^16^H^16^O^3^ | [M+H]^+^ | 257.1172 | 257.1161 | 9.58 | -4.24 | POS |  | I |
| 64 | Biochanin A^[9,10]^ | C_16_H_12_O_5_ | [M+H]^+^ | 285.0757 | 285.0761 | 7.06 | 1.07 | POS | 285.0748 | E |
| 65 | Alpinetin^[9,10]^ | C_16_H_14_O_4_ | [M+H]^+^ | 271.0965 | 271.0964 | 7.1 | -0.28 | POS | 167.0334,271.0956 | E |
| 66 | Isorhamnetin^[9,10]^ | C_16_H_12_O_7_ | [M-H]^-^ | 315.051 | 315.0505 | 7.14 | -1.79 | NEG | 300.0269,315.0507 | E |
| 67 | Isoliquiritigenin^[1]^ | C_15_H_12_O_4_ | [M+FA-H]^-^ | 301.0719 | 301.071 | 7.43 | -2.9 | NEG |  | E |
| 68 | Paeonol^[1]^ | C_9_H_10_O_3_ | 2[M-H]^-^ | 331.1187 | 331.1179 | 7.47 | -2.53 | NEG |  | H |
| 69 | Dalbergin^[1]^ | C_16_H_12_O_4_ | [M-H]^-^ | 267.0663 | 267.0656 | 7.83 | -2.62 | NEG | 206.9867,226.9924,247.0006,252.0421,266.984,267.0658 | I |
| 70 | Ethyl ferulic acid^[1]^ | C_12_H_14_O_4_ | [M-H]^-^ | 221.0819 | 221.0815 | 7.87 | -2.01 | NEG | 134.0362,147.0287,149.0965,154.9917,177.0914,181.0406,193.0875,220.9835,221.0199,221.0816 | I |
| 71 | Medicarpin^[1]^ | C_16_H_14_O_4_ | [M+H]^+^ | 271.0965 | 271.0954 | 8.57 | -4.1 | POS | 123.0438,137.0594,161.0594,271.0955 | H |
| 72 | Odoriflavene^[1]^ | C_17_H_16_O_5_ | [M+H]^+^ | 301.1071 | 301.1057 | 8.65 | -4.59 | POS | 135.0438,137.0595,151.075,153.0544,163.0386,163.0744,273.1113,283.096,301.106 | E |
| 73 | Pinocembrin^[2]^ | C_15_H_12_O_4_ | [M-H]^-^,2[M-H]^-^ | 255.0663 | 255.0657 | 8.89 | -2.17 | NEG | 240.0785,255.0659 | E |
| 74 | Galangin^[2]^ | C_15_H_10_O_5_ | [M-H]^-^ | 269.0455 | 269.0452 | 8.99 | -1.29 | NEG | 228.9884,248.9987,268.9867,269.0453 | E |
| 75 | Oroxylin A^[2]^ | C_16_H_12_O_5_ | [M-H]^-^ | 283.0612 | 283.0607 | 9.13 | -1.82 | NEG | 268.0372,283.0606 | E |
| 77 | Kaempferide^[2]^ | C_16_H_12_O_6_ | [M-H]^-^ | 299.0561 | 299.0556 | 9.21 | -1.69 | NEG | 238.9918,255.0298,256.0371,258.9994,269.0449,278.9850,284.0321,284.0679,298.9940,299.0555 | E |
| 76 | Nerol^[1]^ | C_10_H_18_O | [M+H-H_2_O]^+^ | 137.1324 | 137.1319 | 9.21 | -3.98 | POS | 57.07,67.0542,69.0698,81.0698,95.0854,109.1011,137.0594,137.1323 | L |
| 78 | Rhamnocitrin^[2]^ | C_16_H_12_O_6_ | [M-H_2_O-H]^-^ | 281.0455 | 281.0452 | 9.29 | -1.27 | NEG |  | E |
| 79 | 8-Prenylnaringenin^[2]^ | C_20_H_20_O_5_ | [M+H-H_2_O]^+^ | 323.1277 | 323.1262 | 9.46 | -4.63 | POS | 105.0697,133.0646,135.0438,161.0593,186.067,201.0906,291.1006,323.1263 | E |
| 80 | Tangeretin^[9]^ | C_20_H_20_O_7_ | [M+H]^+^,[M+Na]^+^ | 373.1282 | 373.1267 | 9.57 | -4.05 | POS | 193.0855,343.08,358.1036,373.1271 | E |
| 81 | Danshenxinkun A^[2]^ | C_18_H_16_O_4_ | [M+H-H_2_O]^+^,[M+H]^+^ | 297.1121 | 297.1111 | 9.6 | -3.52 | POS | 233.0956,261.0903,279.1014,279.1364,297.1109 | K |
| 82 | (Rac)-Myrislignan^[10]^ | C_21_H_26_O_6_ | [M+H-H_2_O]^+^,[M+NH_4_]^+^,[M+Na]^+^,[M+K]^+^ | 397.1622 | 397.1608 | 9.73 | -3.67 | POS | 133.0646,165.0908,193.0856 | H |
| 83 | Myrislignan^[10]^ | C_21_H_26_O_6_ | [M-H]^-^ | 373.1657 | 373.1652 | 9.75 | -1.12 | NEG |  | I |
| 84 | Atractylenolide III^[10]^ | C_15_H_20_O_3_ | [M-H]^-^ | 247.134 | 247.1336 | 9.75 | -1.62 | NEG |  | L |
| 85 | Isolan^[10]^ | C_12_H_16_O_3_ | [M-H]^-^ | 207.1027 | 207.1019 | 9.77 | -3.7 | NEG |  | K |
| 86 | Phenethyl ferulate^[10]^ | C_18_H_18_O_4_ | [M+H]^+^ | 299.1278 | 299.1269 | 9.8 | -2.9 | POS | 207.1163,217.101,235.1111,263.1058,281.1163,298.3069,299.126 | I |
| 87 | Ethyl p-methoxycinnamic acid^[10]^ | C_12_H_14_O_3_ | [M+H-H_2_O]^+^,[M+H]^+^ | 207.1016 | 207.101 | 9.81 | -2.63 | POS | 91.0544,119.0491,146.0725,161.0602,161.0959,171.0801,174.0671,189.0906 | I |
| 88 | trans-Methylisoeugenol^[9]^ | C_11_H_14_O_2_ | [M+H-H_2_O]^+^ | 161.0961 | 161.0959 | 9.83 | -1.05 | POS |  | I |
| 89 | Ethyl cinnamate^[9]^ | C_11_H_12_O_2_ | [M+H]^+^ | 177.091 | 177.0906 | 9.86 | -2.37 | POS |  | I |
| 90 | Przewaquinone A^[9]^ | C_19_H_18_O_4_ | [M+H-H_2_O]^+^ | 293.1172 | 293.1163 | 10.09 | -3.07 | POS | 247.1111,275.106,278.0926,293.1159 | K |
| 91 | 6-Prenylapigenin^[9]^ | C_20_H_18_O_5_ | [M-H]^-^ | 337.1081 | 337.1077 | 10.1 | -1.25 | NEG | 256.9835,264.0786,276.9894,293.1180,296.9956,316.9819,316.9884,336.9865,336.9952,337.1085 | E |
| 92 | 4,4'-Dimethoxychalcone^[9]^ | C_17_H_16_O_3_ | [M+FA-H]^-^ | 313.1082 | 313.1079 | 10.1 | -0.86 | NEG |  | E |
| 93 | 2(S)-6-Prenylnaringenin^[8]^ | C_20_H_20_O_5_ | [M+H]^+^ | 341.1384 | 341.1374 | 10.16 | -2.7 | POS |  | E |
| 94 | Moslosooflavone^[8]^ | C_17_H_14_O_5_ | [M+H]^+^ | 299.0914 | 299.0906 | 10.17 | -2.71 | POS |  | E |
| 95 | Dihydrotanshinone I^[9]^ | C_18_H_14_O_3_ | [M+H]^+^,[M+Na]^+^ | 279.1016 | 279.1008 | 10.17 | -2.88 | POS | 233.0956,261.0903,279.1007 | K |
| 96 | 3,4-Dimethoxy-2'-Hydroxychalcone^[1]^ | C_17_H_16_O_4_ | [M+H]^+^ | 285.1121 | 285.1113 | 10.31 | -2.99 | POS | 137.0594,161.0592,285.1113 | E |
| 97 | Pogostone^[1]^ | C_12_H_16_O_4_ | [M+H]^+^ | 225.1121 | 225.1116 | 10.37 | -2.43 | POS | 151.075,162.0671,165.0906,167.0699,168.0775,192.0778,193.0855,210.0881,225.1115 | G |
| 98 | Neocryptotanshinone^[1]^ | C_19_H_22_O_4_ | [M+H]^+^,[M+Na]^+^ | 315.1591 | 315.1582 | 10.4 | -2.75 | POS | 251.1424,279.1371,297.1476,315.1578 | K |
| 99 | Isoimperatorin^[1]^ | C_16_H_14_O_4_ | [M-H_2_O-H]^-^ | 251.0713 | 251.0708 | 10.52 | -1.99 | NEG |  | I |
| 100 | Licarin A^[1]^ | C_20_H_22_O_4_ | [M+H]^+^ | 327.1591 | 327.1582 | 10.65 | -2.75 | POS | 137.0594,143.0851,151.0751,163.0749,171.0801,188.0827,203.1062,286.1192,327.1581 | I |
| 101 | Bavachromene^[2]^ | C_20_H_18_O_4_ | [M+H]^+^ | 323.1278 | 323.1272 | 10.73 | -1.79 | POS | 105.0698,133.0647,161.0595,186.0672,201.0908,323.127 | E |
| 102 | Malabaricone B^[2]^ | C_21_H_26_O4 | [M-H]^-^ | 341.1758 | 341.175 | 10.81 | -2.45 | NEG | 109.0282,341.1752 | H |
| 103 | Tanshinaldehyde^[2]^ | C_19_H_18_O_4_ | [M+FA-H]^-^ | 355.1188 | 355.1181 | 10.83 | -1.92 | NEG | 340.0945,355.1177 | G |
| 104 | Tanshinone I^[2]^ | C_18_H_12_O_3_ | [M+H]^+^,[M+Na]^+^ | 277.0859 | 277.0854 | 10.89 | -1.83 | POS | 249.0902,277.0851 | L |
| 105 | Cryptotanshinone^[2]^ | C_19_H_20_O_3_ | [M+Na]^+^,[M+K]^+^,[M+H]^+^ | 297.1485 | 297.1476 | 10.93 | -3.17 | POS | 319.1293 | K |
| 107 | Gancaonin I^[9]^ | C_21_H_22_O_5_ | [M+H]^+^ | 355.154 | 355.1533 | 10.96 | -2.11 | POS | 133.0645,165.0907,193.0856 | H |
| 106 | Gomisin M2^[2]^ | C_22_H_26_O_6_ | [M+H-H_2_O]^+^ | 369.1696 | 369.1692 | 10.96 | -1.17 | POS |  | I |
| 108 | Schisanhenol^[2]^ | C_23_H_30_O_6_ | [M+Na]^+^ | 425.1935 | 425.1925 | 10.97 | -2.42 | POS | 216.0752,425.1924 | I |
| 109 | beta-Ionone^[10]^ | C_13_H_20_O | [M+H-H_2_O]^+^ | 175.1481 | 175.148 | 11.01 | -0.5 | POS | 87.0442,88.0217,105.0701,119.0857,130.032,133.1013,147.1166,175.075,175.1131,175.1482 | L |
| 110 | Alismol^[1]^ | C_15_H_24_O | [M+H-H_2_O]^+^ | 203.1794 | 203.1793 | 11.01 | -0.43 | POS | 105.0697,107.0853,109.1011,119.0853,121.1010,133.1009,147.1166,161.1321,175.1477,203.1790 | L |
| 111 | Schisanhenol B^[1]^ | C_22_H_26_O_6_ | [M-H]^-^ | 385.1657 | 385.1649 | 11.17 | -1.87 | NEG | 112.9841,137.0227,384.9907,385.1647 | I |
| 112 | Neoprzewaquinone A^[1]^ | C_36_H_28_O_6_ | [M+NH_4_]^+^ | 574.2224 | 574.2227 | 11.2 | 0.59 | POS |  | K |
| 113 | Galbacin^[2]^ | C_20_H_20_O_5_ | [M+H-H_2_O]^+^ | 323.1278 | 323.1273 | 11.26 | -1.51 | POS | 135.0439,149.0595,163.0748,201.0908,323.1271 | I |
| 114 | Schisandrin A^[2]^ | C_24_H_32_O_6_ | [M+H]^+^ | 417.2272 | 417.2269 | 11.49 | -0.69 | POS |  | I |
| 115 | Tanshinone IIA^[1-10]^ | C_19_H_18_O_3_ | [M+H]^+^,[M+Na]^+^,[M+K]^+^,[M+NH_4_]^+^,[M+H-H_2_O]^+^ | 295.1329 | 295.1325 | 11.62 | -1.35 | POS | 249.1268,277.1216,295.132 | L |
| 116 | Miltirone^[1,2]^ | C_19_H_22_O_2_ | [M+H]^+^,[M+Na]^+^ | 283.1693 | 283.1686 | 11.83 | -2.26 | POS | 223.1115,240.114,265.1581,283.169 | L |
| 117 | Corilagin^[1,2]^ | C_27_H_22_O_18_ | [M+H-H_2_O]^+^ | 617.0774 | 617.0795 | 12.14 | 3.38 | POS |  | H |
| 118 | alpha-Linolenic acid^[1,2]^ | C_18_H_30_O_2_ | [M-H]^-^,[M+FA-H]^-^ | 277.2173 | 277.2169 | 12.2 | -1.52 | NEG | 277.2173 | D |

Note A: Alkaloids;B: Amino Acids, Peptides and derivatives; C: Carbohydrates and Glycosides; D: Fatty Acyls; E: Flavonoids; F: Organic acids and derivatives; G: Others; H: Phenols; I: Phenylpropanoids; J: Pyridines and derivatives; K: Quinones; L:Terpenes.

Reference：

[1] TCMIP: <http://www.tcmip.cn/TCMIP/index.php/Home/Login/login.html>.

[2] TCMSP: <https://www.tcmsp-e.com/load_intro.php?id=43>.

[3] Tian,H.X., Yang, R.Q., ZHOU, H.Q.(2021). High-speed identification of odor changes and substance basis of Myristicae Semen mildew by electronic nose and HS-GC-MS. *China Journal of Chinese Materia Medica*, 46(22), 5853-5860.

[4] Liu, Y., Ding, X. Y., Wang, N.(2024). Component characterization of Salvia miltiorrhiza extracts based on UPLC-QExactive Plus-Orbitrap MS and analysis of pharmacodynamic material basis related to spectral effect of its antithrombotic activity. *Chinese Traditional and Herbal Drugs*, 55(05), 1609-1619.

[5] Zhang, N. N., Wang, W. W., Xu, D. P.(2009). Determination of Bioactive Components of Benzene/Ethanol Extractives from Santalum album Leaves by Py-GC/MS. *Journal of Central South University of Forestry & Technology*, 29(04),70-73.

[6] Zhang, G. Z.(2007). Analysis of Constituents of Essential Oils in Rhizoma Kaempferiae by GC-MS. *Asia-Pacific Traditional Medicine*, (07), 56-59.

[7] Meng, X. W., Zhu, Q., Zhang, N.(2020). Rapid Identification on Chemical Components in Dalbergiae Odoriferae Lignum by UPLC-Q-TOF-MS/MS. *Chinese Journal of Experimental Traditional Medical Formulae*, 26(10), 107-118.

[8]Yang, Z. F., Wang, Q.,Zhao, Q.(2024). Identification and Characterization of Flavonoid Compounds from Hippophae rhamnoides L.by UPLC-Q-Orbitrap MS. *Chemical Reagents*, 46(02), 95-106.

[9] Liao, Y. H.,Lu, J. K., Niu, Y. (2025). Study on anti-atherosclerosis mechanism of blood components of Guanxin Qiwei tablets based on HPLC-Q-Exactive-MS/MS and network pharmacology. *Acta Pharmaceutica Sinica*, 60(02), 449-458.

[10] Liu, C. D., Guo, J. Y., Li, J.(2024). Qualitative analysis of chemical constituents of Guanxin Qiwei tablets based on HPLC-Q-Exactive-MS technique. *Central South Pharmacy*, 22(03), 691-698.
